# Supplementary material for: In Vitro/In Vivo Translation of Synergistic Combination of MDM2 and MEK Inhibitors in Melanoma Using PBPK/PD Modelling: Part II
Source: Int J Mol Sci. 2022 Oct 8;23(19):11939. doi: 10.3390/ijms231911939 (PMC9570053; doi:10.3390/ijms231911939)
Supplement: Supplementary file 1 [file ijms-23-11939-s001.zip › ijms-1906160-supplementary.pdf]

International Journal of *Molecular Sciences*

MDPI

Supplementary Materials

In Vitro/In Vivo Translation of Synergistic Combination of MDM2 and MEK Inhibitors in Melanoma Using PBPK/PD Modelling: Part II

Jakub Witkowski, Sebastian Polak, Zbigniew Rogulski and Dariusz Pawelec

Table S1. Siremadlin and trametinib resistant population estimates and pharmacodynamic interaction parameters [1].

Compound	% of viability at last observation ± SD	Synergyfinder % Mean score δ ± SD	Synergy % β parameter ± SD
Siremadlin	21.445 ± 6.389	-	-
Trametinib	14.097 ± 2.594	-	-
Siremadlin + Trametinib		7.48 ± 1.88	23.13 ± 4.80

Table S2. Differences in PBPK model parameters with and without interaction at PK level and related to administration in various formulations.

Drug/ Parameter	ka (1//h)	tlag (h)	fa (%)	Tumour PS (ml/min/ml)	Tumour P-gp efflux transporter CL_{int} (ml/min/ml)	Notes
Siremadlin	0.31	0	0.6023	0.084656	95.54	-
Siremadlin (literature)	3.77	0.73	0.6607	-	-	fa optimized from range 0.5972-0.6607 (calculated from P.O. and I.V. data [2,3])
Siremadlin (PK interaction)	0.65	-	-	0.1407	47.12	-
Trametinib	1.25	0.85	0.7247	0.02482	60.27	-
Trametinib (literature)	0.87	0	0.7796	-	-	fa optimized from range 0.6893-0.7796 (calculated from P.O. and I.V. data from [4,5])
Trametinib (PK interaction)	0.357	0	0.6407	0.11092	214.43	-

ka: absorption rate constant. *tlag*: lag time. *fa*: fraction of dose absorbed. *Tumour PS*: Passive permeability clearance between intra- and extracellular water of tumour. *Tumour P-gp efflux transporter* CL_{int}: *In vitro* transporter mediated intrinsic clearance in tumour.

Table S3. Comparison of predicted vs observed key PK parameters (AUC/Cmax/Tmax) for siremadlin.

Tissue	AUC_{obs}	AUC_{pred}	AUC_{pred}/AUC_{obs}	$C_{max,obs}$	$C_{max,pred}$	$C_{max,pred}/C_{max,obs}$	$T_{max,obs}$	$T_{max,pred}$	$T_{max,pred}/T_{max,obs}$
Plasma	95092.97	88087.91	0.93	9777.67	10752.58	1.10	1.50	2.77	1.85
A375 tumour	179026.48	218705.89	1.22	16214.30	16214.30	1.00	1.50	6.40	4.26
Muscle	170157.96	157590.47	0.93	21631.93	19189.57	0.89	1.50	3.00	2.00
Spleen	211232.59	195660.09	0.93	25195.17	23881.49	0.95	1.50	2.82	1.88
Brain	8942.01	8280.25	0.93	773.94	1010.74	1.31	1.50	2.77	1.85
Heart	286296.23	265203.84	0.93	36316.90	32372.64	0.89	1.50	2.77	1.85
Kidney	353015.40	327004.35	0.93	40979.10	39916.46	0.97	1.50	2.78	1.86
Skin	271818.44	251741.80	0.93	19523.67	30669.64	1.57	1.50	2.98	1.98
Lung	197152.26	182632.35	0.93	22627.91	22293.34	0.99	1.50	2.77	1.85
Gut	608367.79	563442.34	0.93	59509.80	68644.24	1.15	4.00	2.98	0.74
Liver	479296.64	444154.33	0.93	59431.53	55581.49	0.94	1.50	2.28	1.52

Table S4. Comparison of predicted vs observed key PK parameters (AUC/Cmax/Tmax) for trametinib.

Tissue	AUC_{obs}	AUC_{pred}	AUC_{pred}/AUC_{obs}	$C_{max,obs}$	$C_{max,pred}$	$C_{max,pred}/C_{max,obs}$	$T_{max,obs}$	$T_{max,pred}$	$T_{max,pred}/T_{max,obs}$
Plasma	5580.83	5213.47	0.93	567.02	626.09	1.10	4.00	2.64	0.66
A375 tumour	9131.17	8729.04	0.96	587.25	587.25	1.00	4.00	3.30	0.83
Muscle	5725.86	5345.50	0.93	420.58	641.14	1.52	1.50	2.78	1.86
Spleen	14596.18	13631.00	0.93	930.88	1636.79	1.76	1.50	2.70	1.80
Brain	1026.55	958.70	0.93	55.48	115.14	2.08	24.00	2.64	0.11
Heart	7542.96	7046.14	0.93	639.78	846.22	1.32	1.50	2.64	1.76
Kidney	18204.41	17005.08	0.93	1471.71	2042.25	1.39	1.50	2.66	1.78
Skin	6377.44	5954.98	0.93	515.13	714.92	1.39	4.00	2.72	0.68
Lung	7367.20	6880.24	0.93	752.50	826.47	1.10	4.00	2.64	0.66
Gut	30558.37	28521.48	0.93	3044.37	3415.76	1.12	1.50	2.84	1.90
Liver	30111.52	28146.52	0.93	2356.01	3393.93	1.44	4.00	2.50	0.62

Table S5. Comparison of predicted vs observed key PK parameters (AUC/Cmax/Tmax) for siremadlin with interaction with trametinib.

Tissue	AUC_{obs}	AUC_{pred}	AUC_{pred}/AUC_{obs}	$C_{max,obs}$	$C_{max,pred}$	$C_{max,pred}/C_{max,obs}$	$T_{max,obs}$	$T_{max,pred}$	$T_{max,pred}/T_{max,obs}$
Plasma	107993.98	88326.59	0.82	14559.95	15232.78	1.05	1.50	1.78	1.18
A375 tumour	218677.07	335953.22	1.54	28613.74	28613.67	1.00	1.50	5.08	3.38
Muscle	207541.97	158044.40	0.76	30385.46	27093.64	0.89	1.50	2.02	1.34
Spleen	231909.42	196197.92	0.85	37783.50	33826.65	0.90	1.50	1.82	1.22
Brain	10637.20	8302.71	0.78	1399.80	1431.88	1.02	1.50	1.78	1.18
Heart	321668.15	265924.65	0.83	48642.34	45860.83	0.94	1.50	1.79	1.19
Kidney	446226.08	327894.16	0.73	67620.00	56547.47	0.84	1.50	1.79	1.19
Skin	317510.56	252462.44	0.80	33168.04	43332.58	1.31	1.50	1.99	1.33
Lung	239204.08	183126.99	0.77	36854.11	31582.12	0.86	1.50	1.78	1.18
Gut	654824.01	565055.19	0.86	97109.13	96986.58	1.00	1.50	1.99	1.33
Liver	582012.28	445220.68	0.76	95121.30	81889.52	0.86	1.50	1.21	0.81

Table S6. Comparison of predicted vs observed key PK parameters (AUC/Cmax/Tmax) for trametinib with interaction with siremadlin.

Tissue	AUC_{obs}	AUC_{pred}	AUC_{pred}/AUC_{obs}	$C_{max,obs}$	$C_{max,pred}$	$C_{max,pred}/C_{max,obs}$	$T_{max,obs}$	$T_{max,pred}$	$T_{max,pred}/T_{max,obs}$
Plasma	4484.99	4554.43	1.02	353.65	390.02	1.10	4.00	4.03	1.01
A375 tumour	9656.67	11739.16	1.22	714.53	714.53	1.00	4.00	7.57	1.89
Muscle	5713.07	4668.55	0.82	456.60	399.94	0.88	4.00	4.15	1.04
Spleen	13530.74	11906.40	0.88	764.55	1019.89	1.33	4.00	4.07	1.02
Brain	1028.61	837.56	0.81	55.48	71.73	1.29	24.00	4.03	0.17
Heart	7870.39	6155.72	0.78	753.69	527.17	0.70	4.00	4.03	1.01
Kidney	17234.73	14855.28	0.86	1324.75	1272.21	0.96	4.00	4.03	1.01
Skin	6882.91	5201.36	0.76	633.49	445.57	0.70	4.00	4.09	1.02
Lung	7656.70	6014.07	0.79	846.31	515.33	0.61	4.00	4.03	1.01
Gut	29204.64	24907.32	0.85	2779.56	2133.50	0.77	1.50	4.19	2.79

Tissue	AUC_{obs}	AUC_{pred}	AUC_{pred}/AUC_{obs}	$C_{max,obs}$	$C_{max,pred}$	$C_{max,pred}/C_{max,obs}$	$T_{max,obs}$	$T_{max,pred}$	$T_{max,pred}/T_{max,obs}$
Liver	30547.92	24593.83	0.81	2486.85	2107.07	0.85	4.00	3.89	0.97

Table S7. Parameters of the PBPK model for siremadlin.

Model Section	Parameter (Units)	Value	Source/Reference/Comments
**Physiochemical properties and blood binding**	Molecular Weight (g/mol)	555.41	-
	logP	2.99	*In vitro* determined - Unpublished Adamed Pharma data (value similar to reported 2.90 [6])
	Compound Type	Monoprotic Base	-
	pKa	1.69	*In vitro* determined - Unpublished Adamed Pharma data
	B/P	0.76	*In vitro* determined - Unpublished Adamed Pharma data (arithmetic mean from 2.5-10uM - range 0.66-0.86)
	fu plasma	0.0966	*In vitro* determined - Unpublished Adamed Pharma data (arithmetic mean from 2.5-10uM range after 18h of incubation which was needed to reach equilibrium – range 0.0707-0.1113)
**Absorption**	Absorption model	First-Order	-
	fa	0.60231	Optimized from range 0.5697-0.6303 (calculated from Adamed Pharma P.O. data and I.V. data from [3])
	ka (1/h)	0.31	Optimized
	Lag time (h)	0	Optimized
**Distribution**	Distribution model	Full PBPK	-
	Vss (L/kg)	2.802	Simcyp predicted (Method 3) – value similar to the reported data range 2.456-3.232 [3]
	Smoothing function	Enabled	-
	Sub-Cellular Distribution model	Enabled	Only for Adipose and Bone tissues

Model Section	Parameter (Units)	Value	Source/Reference/Comments
	Olive oil:water partition as a surrogate for neutral lipid partition option	Disabled	-
	Kp Brain	0.094	Calculated according to Equation 1 based on Adamed Pharma data
	Kp Gut	6.3976	Calculated according to Equation 1 based on Adamed Pharma data
	Kp Heart	3.0107	Calculated according to Equation 1 based on Adamed Pharma data
	Kp Kidney	3.7123	Calculated according to Equation 1 based on Adamed Pharma data
	Kp Liver	5.0403	Calculated according to Equation 1 based on Adamed Pharma data
	Kp Lung	2.0733	Calculated according to Equation 1 based on Adamed Pharma data
	Kp Muscle	1.7894	Calculated according to Equation 1 based on Adamed Pharma data
	Kp Skin	2.8584	Calculated according to Equation 1 based on Adamed Pharma data
	Kp Spleen	2.2213	Calculated according to Equation 1 based on Adamed Pharma data
	Kp Scalar	1.4478	Optimized
**Elimination**	Clearance type	Mouse Hepatocytes	-
	Hep intrinsic CL (mL/min/10^6 cells)	18.15	Optimized from range 18.15-18.58 (In vitro determined - Unpublished Adamed Pharma data)
	fu_inc	0.54	Optimized from range 0.52-0.64 (In vitro determined - Unpublished Adamed Pharma data)
**Tumour**	Tumour model type	Permeability-limited tumour model	-

Model Section	Parameter (Units)	Value	Source/Reference/Comments
	Tumour PS (mL/min/mL of tumour volume)	0.084656	Manual optimization
	Tumour P-gp efflux transporter CL_{int} (mL/min mL tumour)	95.54	Manual optimization
	fu_{EW}	0.076	Manual optimization
	fu_{IC}	0.005	Manual optimization
**Trial Design**	Administration route	Oral	-
	Dose (mg/kg)	100	-
	Dose interval τ (h)	24	-
	Condition	Fasted	--
	Simulation duration	24h<br>72h	Single administration<br>Multiple administration

B/*P:* blood to plasma partition ratio. *fu plasma:* fraction unbound in plasma. *fa*: fraction of dose absorbed. *ka*: absorption rate constant. *Vss*: volume of distribution at steady-state. *Kp*: tissue-to-plasma partition coefficient. Hep: Hepatocytes. *CL*: clearance. *fu_inc*: fraction of unbound drug in the *in vitro* system. Tumour *PS*: Passive permeability clearance between intra- and extracellular water of tumour. *Tumour P-gp efflux transporter* CL_{int}: *In vitro* transporter mediated intrinsic clearance in tumour. fu_{EW}*:* fraction unbound in the extracellular water of tumour. Fu_{IC}*:* fraction unbound in the intracellular water of tumour.

Table S8. Parameters of the PBPK model for trametinib.

Model Section	Parameter (Units)	Value	Source/Reference/Comments
**Physiochemical properties and blood binding**	Molecular Weight (g/mol)	615.39	-
	logP	4.10	*In vitro* determined - Unpublished Adamed Pharma data

Model Section	Parameter (Units)	Value	Source/Reference/Comments
	Compound Type	Monoprotic Base	-
	pKa	11.15	*In vitro* determined - Unpublished Adamed Pharma data
	B/P	0.70	*In vitro* determined - Unpublished Adamed Pharma data (arithmetic mean from 0.81uM – range – 0.68-0.72 [7]
	fu plasma	0.05	[7]
**Absorption**	Absorption model	First-Order	-
	fa	0.7247	Optimized from range 0.6407-0.7247 (calculated from Adamed Pharma P.O. data and I.V. data from [5])
	ka (1/h)	1.25	Optimized
	Lag time (h)	0.85	Optimized
**Distribution**	Distribution model	Full PBPK	-
	Vss (L/kg)	1.35	Simcyp predicted (Method 3) – value similar to the reported 0.9L/kg [7]
	Smoothing function	Enabled	-
	Sub-Cellular Distribution model	Enabled	Only for Adipose and Bone tissues
	Olive oil:water partition as a surrogate for neutral lipid partition option	Disabled	-
	Kp Brain	0.1839	Calculated according to Equation 1 based on Adamed Pharma data
	Kp Gut	5.4756	Calculated according to Equation 1 based on Adamed Pharma data
	Kp Heart	1.3516	Calculated according to Equation 1 based on Adamed Pharma data
	Kp Kidney	3.2620	Calculated according to Equation 1 based on Adamed Pharma data

Model Section	Parameter (Units)	Value	Source/Reference/Comments
	Kp Liver	5.3955	Calculated according to Equation 1 based on Adamed Pharma data
	Kp Lung	1.3201	Calculated according to Equation 1 based on Adamed Pharma data
	Kp Muscle	1.0260	Calculated according to Equation 1 based on Adamed Pharma data
	Kp Skin	1.1427	Calculated according to Equation 1 based on Adamed Pharma data
	Kp Spleen	2.6154	Calculated according to Equation 1 based on Adamed Pharma data
	Kp Scalar	0.0276	Optimized
**Elimination**	Clearance type	I.V. clearance	-
	CL_{iv} (mL/min)	0.096425	Similar to the previously reported value 3.5 mL/min/kg [7]
**Tumour**	Tumour model type	Permeability-limited tumour model	-
	Tumour PS (mL/min/mL of tumour volume)	0.02482	Manual optimization
	Tumour P-gp efflux transporter CL_{int} (mL/min mL tumour)	60.27	Manual optimization
	fu_{EW}	0.016	Manual optimization
	fu_{IC}	0.0025	Manual optimization
**Trial Design**	Administration route	Oral	-

Model Section	Parameter (Units)	Value	Source/Reference/Comments
	Dose (mg/kg)	1	-
	Dose interval τ (h)	24	-
	Condition	Fasted	--
	Simulation duration	24h<br>144h	Single administration<br>Multiple administration

B/P: blood to plasma partition ratio. *fu plasma:* fraction unbound in plasma. *fa*: fraction of dose absorbed. *ka*: absorption rate constant. *Vss*: volume of distribution at steady-state. *Kp*: tissue-to-plasma partition coefficient. Hep: Hepatocytes. *CL*: clearance. *fu_inc*: fraction of unbound drug in the *in vitro* system. Tumour *PS*: Passive permeability clearance between intra- and extracellular water of tumour. *Tumour P-gp efflux transporter* CL_{int}: *In vitro* transporter mediated intrinsic clearance in tumour. fu_{EW}: fraction unbound in the extracellular water of tumour. Fu_{IC}: fraction unbound in the intracellular water of tumour.

Table S9. Parameters of the PD (TGI) models for single administration of siremadlin, trametinib and their combination in the current study. Models outcomes are depicted in Figures 3-4 and S4.

Compound/ Parameter	Description	Vehicle	Siremadlin	Trametinib	Siremadlin+Trametinib
Dose (mg/kg)	-	-	40/100	0.3/1	40+0.3/40+1/100+0.3/100+1
Administration schedule	-	qdx6	qdx3	qdx6	qdx3/qdx6
Score	-	-519	-103/-145	-135/-156	-96/-142/-152/-176
TS0	initial tumour size sensitive population (mL)	0.1628	0.1723/0.1635	0.1796/0.1671	0.1699/0.1663/0.1655/0.1681
TSr0	initial tumour size resistant population (mL)	0.000001	0.000001	0.000001	0.000001
crck	initial tumour size and max tumour size correlation constant	15.027	12.50/9.90	6.70/10.60	16.10/9.60/10.20/8.75

kge	Tumour growth rate (1/day)	0.005782	0.0072	0.0063/0.0075	0.0063/0.0075/0.0063/0.0075
s	Killing constant coefficient	-	0.3	0.3	0.3
lambda	Resistance factor	-	7	5	12
tau	Effect delay (h)	-	10/11	4.5/0.5	5.5/9.5/6.5/10.5
kkill	Tumour killing constant	-	0.0086/0.0215	0.00705/0.0235	-
ksr	Sensitive to resistant cells conversion rate (%)	-	0.2145	0.141	0.03024
gamma	Drug interaction β parameter (%)	-	-	-	1.2313
AUC ratio Siremadlin	Exposure ratio in combination	-	-	-	1.74/1.00/1.06/1.14
AUC ratio Trametinib	Exposure ratio in combination	-	-	-	1.18/0.70/0.82/0.80
Mean Relative Error (%)	-	3.44	8.23/11.71	6.00/13.89	19.98/13.45/18.94/14.16

Table S10. Parameters of the PD (TGI) models for siremadlin in verification datasets. Models outcome is depicted in Figure S5.

Compound/ Parameter	Description	Vehicle	Siremadlin	Siremadlin
Dose (mg/kg)	-	-	25/50	50/100
Administration schedule	-	qdx5/ q7dx2	qdx5	q7dx2

Source	-	Adamed Pharma	Adamed Pharma	Adamed Pharma
Score	-	-70/-60	-58/-44	-57/-41
TS0	initial tumour size sensitive population (mL)	0.1354/0.1355	0.1368/0.1376	0.1362/0.1375
TSr0	initial tumour size resistant population (mL)	0.000001	0.000001	0.000001
crck	initial tumour size and max tumour size correlation constant	89.000/88.901	20.0/34.0	12.5/10.0
kge	Tumour growth rate (1/day)	0.00499/0.00537	0.0072	0.0072
s	Killing constant coefficient	-	0.3	0.3
lambda	Resistance factor	-	7	7
tau	Effect delay (h)	-	0/2.13	2.117/5.025
kkill	Tumour killing constant	-	0.0090/0.0179	0.0072/0.0143
ksr	Sensitive to resistant cells conversion rate (%)	-	0.2145	0.2145
AUC ratio Siremadlin	Exposure ratio	-	0.625	0.625
Mean Relative Error (%)	-	2.76/3.33	4.43/8.17	5.23/4.15

Table S11. Parameters of the PD (TGI) models for trametinib in verification datasets. Models outcome is depicted in Figure S6.

**Compound/ Parameter**	**Description**	**Vehicle**	**Trametinib**	**Vehicle**	**Trametinib**

Dose (mg/kg)	-	-	0.3	-	0.1/0.3/3
Administration schedule	-	qdx19	qdx36	qdx14	qdx14
Source	-	[8]	[8]	[9]	[9]
Score	-	-29	39	-29	-30/-50/203
TS0	initial tumour size sensitive population (mL)	0.2020	0.1854	0.2281	0.2253/0.2329/0.2186
TSr0	initial tumour size resistant population (mL)	0.000001	0.000001	0.000001	0.000001
crck	initial tumour size and max tumour size correlation constant	8.896	14.8	6.468	6.20/4.40/0.87
kge	Tumour growth rate (1/day)	0.00766	0.0081	0.00917	0.0093/0.0097/0.014
s	Killing constant coefficient	-	0.3	-	0.3
lambda	Resistance factor	-	5	-	5
tau	Effect delay (h)	-	5.542	-	25
kkill	Tumour killing constant	-	0.0423	-	0.0055/0.01645/0.1645
ksr	Sensitive to resistant cells conversion rate (%)	-	0.141	-	0.141

AUC ratio Trametinib	Exposure ratio	-	0.328	-	1/1/0.2
Mean Relative Error (%)	-	3.72	5.26	4.96	3.31/4.03/19.72

Table S12. Relationships between universal TGI model parameters for siremadlin, trametinib and its combination. Visualization of relationships between parameters are depicted in Figures S7-S14.

Parameter/ Compound	Vehicle	Siremadlin (HDM)	Trametinib (TRA)	Siremadlin+ Trametinib combination
TSr0	Fixed ~0	Fixed ~0	Fixed ~0	Fixed ~0
TS0	Initial study arm value	Initial study arm value	Initial study arm value	Initial study arm value
crck	Estimated (x)	-0.0506*dose + 14.839 [R^2 = 0.989][1]	(-0.0041+kge)/(0.00033-0.000011*dose) [R^2=1][2]	(-9.153+HDM crck)/(-4.057+0.671*TRA crck)+8.506 [R^2=1][3]
kge	Estimated (x)	1.2457x	0.0017*TRA dose+0.0058 [R^2=0.996][4]	As in longer administered drug/ in case of equal administration time: mean (Siremadlin kge; Trametinib kge)
AUC ratio	-	2.744-1.834*TRA dose-0.017*HDM dose+0.019*HDM dose * TRA dose [R^2=1][5]	-0.000016*TRA dose * (HDM dose)^2+0.00057*(HDM dose)^2 *(TRA dose)^2-0.006*(HDM dose)$^{1.5}$*(TRA dose)$^{1.5}$ +1.36 [R^2=1][6]	-
a	-	0.00007167	-	-
b	-	-	0.003917	-

kkill	-	a * HDM dose * number of doses * exposure ratio	b * TRA dose * number of doses * exposure ratio	(a * HDM dose * number of doses * exposure ratio + b * TRA dose * number of doses * exposure ratio)*gamma
s	-	Estimated (x)	Estimated (y)	Mean (x;y)
lambda	-	Estimated (x)	Estimated (y)	x + y
tau	-	6.8133*HDM dose$^{0.104}$ [R^2=1][7]	(-0.55*TRA dose/(-0.01*TRA dose-1.51*kge+TRA dose*kge))-46.18*TRA dose+7268.45*kge-42.01 [R^2=1][8]	∣HDM tau-TRA tau∣
ksr	-	Estimated (x)	Estimated (y)	x*y
gamma	-	-	-	From *in vitro* synergy package analysis[9]

[1] Relationship between parameters is depicted in Figure S7.
[2] Relationship between parameters is depicted in Figure S8.
[3] Relationship between parameters is depicted in Figure S9.
[4] Relationship between parameters is depicted in Figure S10.
[5] Relationship between parameters is depicted in Figure S11.
[6] Relationship between parameters is depicted in Figure S12.
[7] Relationship between parameters is depicted in Figure S13.
[8] Relationship between parameters is depicted in Figure S14.
[9] See Table S1.

Table S13. Comparison of siremadlin PK parameters between current study and previously performed at Adamed Pharma.

Source	Dose (mg/kg)	Cmax (nM)	Cmax ratio	AUC_{0-24h} (nM x h)	AUC ratio
Current study	100	9778	0.229	95093	0.625
Adamed Pharma	100	42753	1.000	152225	1.000

Table S14. Calculations of the HED doses for siremadlin and trametinib which are currently examined in clinical trials.

Compound	Regimen	Cycle length	Dose range in clinical trials (mg) [10,11]	Human dose in simulation (mg/kg)*	Animal dose equivalent to HED in simulation (mg/kg)*, #	Notes
Siremadlin	qdx21 (1A)	21	0.18-5.00	-	-	
Siremadlin	qwx2 (1B)	28	1.71-2.86	-	-	
Siremadlin	qdx14 (2A)	28	0.01-0.29	-	-	
Siremadlin	qdx7 (2C)	28	0.21-0.36	-	-	
Siremadlin	qdx21 (1A)	21	-	3.69	48.996	Tumour Stasis dose modelled from [12]
Siremadlin	qdx21 (1A)	21	-	5.00	66.468	Maximal tested dose in this regimen
Siremadlin	qwx2 (1B)	28	-	1.71	22.789	Recommended dose for expansion (RDE) [10]
Siremadlin	qwx2 (1B)	28	-	2.46	32.759	Tumour Stasis dose modelled from [12]
Siremadlin	qwx2 (1B)	28	-	2.86	37.982	Maximal tested dose in this regimen
Siremadlin	qdx14 (2A)	28	-	0.29	3.798	Maximal tested dose in this regimen
Siremadlin	qdx7 (2C)	28	-	0.36	4.748	Maximal tested dose in this regimen
Trametinib	Continuous	Up to drug-related grade 2 toxicity	2	0.029	0.3798	Approved dose [5]

* Assumed Human bodyweight 70kg
# Assumed mice body weight 0.02755kg (mean mice weight from drug combination study)

Table S15. Parameters for simulations of tumour volume with the use of universal TGI model at HED doses for single siremadlin administration. Simulation outcomes are depicted in Figures S15-S16.

Siremadlin dose [mg/kg]	Doses in cycle	TSr0	TS0	crck	kge	kkill	s	lambda	tau	ksr
22.789	2	0.000001	0.170	13.69	0.0072	0.0032664	0.3	7	9.43	0.2145
32.759	2	0.000001	0.170	13.18	0.0072	0.0046955	0.3	7	9.79	0.2145
37.982	2	0.000001	0.170	12.92	0.0072	0.0054440	0.3	7	9.95	0.2145
48.996	1	0.000001	0.170	12.36	0.0072	0.0035114	0.3	7	10.21	0.2145
66.468	1	0.000001	0.170	11.48	0.0072	0.0047635	0.3	7	10.54	0.2145
3.798	14	0.000001	0.170	14.65	0.0072	0.0038108	0.3	7	7.83	0.2145
4.748	7	0.000001	0.170	14.60	0.0072	0.0023818	0.3	7	8.01	0.2145

Table S16. Parameters for simulations of tumour volume with the use of universal TGI model at HED doses for single trametinib administration. Simulation outcomes are depicted in Figures S17-S18.

Trametinib dose [mg/kg]	Doses in cycle	TSr0	TS0	crck	kge	kkill	s	lambda	tau	ksr
0.3798	40	0.000001	0.17	7.163	0.00645	0.0595046	0.3	5	4.76	0.141
0.3798	28	0.000001	0.17	7.163	0.00645	0.0416532	0.3	5	4.76	0.141
0.3798	21	0.000001	0.17	7.163	0.00645	0.0312399	0.3	5	4.76	0.141
0.3798	14	0.000001	0.17	7.163	0.00645	0.0208266	0.3	5	4.76	0.141

Table S17. Parameters for simulations of tumour volume with the use of universal TGI model at HED doses for siremadlin and trametinib co-administration. Simulations outcomes are depicted in Figures 5 and S19.

Siremadlin dose [mg/kg]	Trametinib dose [mg/kg]	Sire-madlin doses in cycle	Trametinib doses in cycle	TSr0	TS0	crck	kge	Siremadlin AUC ratio	Trametinib AUC ratio	kkill Siremadlin	kkill Trametinib	s	lambda	tau	ksr	gamma
22.789	0.3798	2	40	0.000001	0.17	14.539	0.00645	1.8240	1.2438	0.003266	0.059505	0.3	12	4.667	0.03024	1.2313
32.759	0.3798	2	40	0.000001	0.17	13.867	0.00645	1.7265	1.1731	0.004695	0.059505	0.3	12	5.029	0.03024	1.2313
37.982	0.3798	2	40	0.000001	0.17	13.516	0.00645	1.6754	1.1345	0.005444	0.059505	0.3	12	5.181	0.03024	1.2313
3.798	0.3798	14	40	0.000001	0.17	15.818	0.00645	2.0098	1.3508	0.003811	0.059505	0.3	12	3.063	0.03024	1.2313
4.748	0.3798	7	40	0.000001	0.17	15.754	0.00645	2.0005	1.3472	0.002382	0.059505	0.3	12	3.247	0.03024	1.2313
22.789	0.3798	2	28	0.000001	0.17	14.539	0.00645	1.8240	1.2438	0.003266	0.041653	0.3	12	4.667	0.03024	1.2313
32.759	0.3798	2	28	0.000001	0.17	13.867	0.00645	1.7265	1.1731	0.004695	0.041653	0.3	12	5.029	0.03024	1.2313
37.982	0.3798	2	28	0.000001	0.17	13.516	0.00645	1.6754	1.1345	0.005444	0.041653	0.3	12	5.181	0.03024	1.2313
3.798	0.3798	14	28	0.000001	0.17	15.818	0.00645	2.0098	1.3508	0.003811	0.041653	0.3	12	3.063	0.03024	1.2313
4.748	0.3798	7	28	0.000001	0.17	15.754	0.00645	2.0005	1.3472	0.002382	0.041653	0.3	12	3.247	0.03024	1.2313
22.789	0.3798	2	21	0.000001	0.17	14.539	0.00645	1.8240	1.2438	0.003266	0.031240	0.3	12	4.667	0.03024	1.2313
32.759	0.3798	2	21	0.000001	0.17	13.867	0.00645	1.7265	1.1731	0.004695	0.031240	0.3	12	5.029	0.03024	1.2313
37.982	0.3798	2	21	0.000001	0.17	13.516	0.00645	1.6754	1.1345	0.005444	0.031240	0.3	12	5.181	0.03024	1.2313
48.996	0.3798	1	21	0.000001	0.17	12.774	0.00645	1.5676	1.0515	0.003511	0.031240	0.3	12	5.448	0.03024	1.2313
66.468	0.3798	1	21	0.000001	0.17	11.597	0.00645	1.3967	0.9204	0.004764	0.031240	0.3	12	5.777	0.03024	1.2313
3.798	0.3798	14	21	0.000001	0.17	15.818	0.00645	2.0098	1.3508	0.003811	0.031240	0.3	12	3.063	0.03024	1.2313
4.748	0.3798	7	21	0.000001	0.17	15.754	0.00645	2.0005	1.3472	0.002382	0.031240	0.3	12	3.247	0.03024	1.2313
22.789	0.3798	2	14	0.000001	0.17	14.539	0.00645	1.8240	1.2438	0.003266	0.020827	0.3	12	4.667	0.03024	1.2313
32.759	0.3798	2	14	0.000001	0.17	13.867	0.00645	1.7265	1.1731	0.004695	0.020827	0.3	12	5.029	0.03024	1.2313
37.982	0.3798	2	14	0.000001	0.17	13.516	0.00645	1.6754	1.1345	0.005444	0.020827	0.3	12	5.181	0.03024	1.2313
48.996	0.3798	1	14	0.000001	0.17	12.774	0.00645	1.5676	1.0515	0.003511	0.020827	0.3	12	5.448	0.03024	1.2313
66.468	0.3798	1	14	0.000001	0.17	11.597	0.00645	1.3967	0.9204	0.004764	0.020827	0.3	12	5.777	0.03024	1.2313
3.798	0.3798	14	14	0.000001	0.17	15.818	0.00682	2.0098	1.3508	0.003811	0.020827	0.3	12	3.063	0.03024	1.2313
4.748	0.3798	7	14	0.000001	0.17	15.754	0.00645	2.0005	1.3472	0.002382	0.020827	0.3	12	3.247	0.03024	1.2313

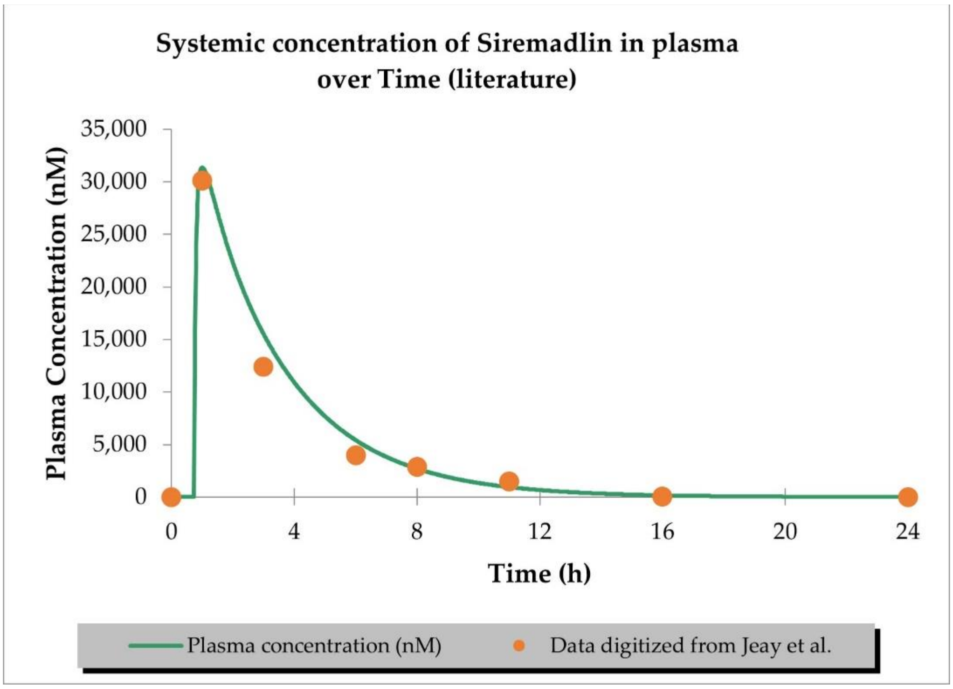

Figure S1. PBPK model of siremadlin verified with literature data (data digitized from Jeay et al. [2]).

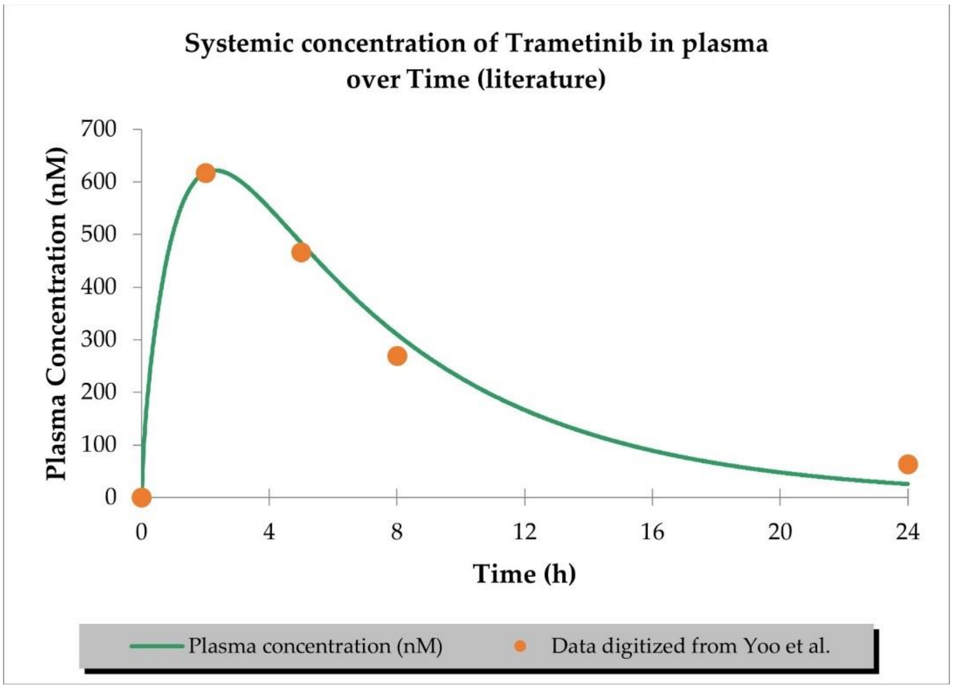

Figure S2. PBPK model of trametinib verified with literature data (data digitized from Yoo et al. [4]).

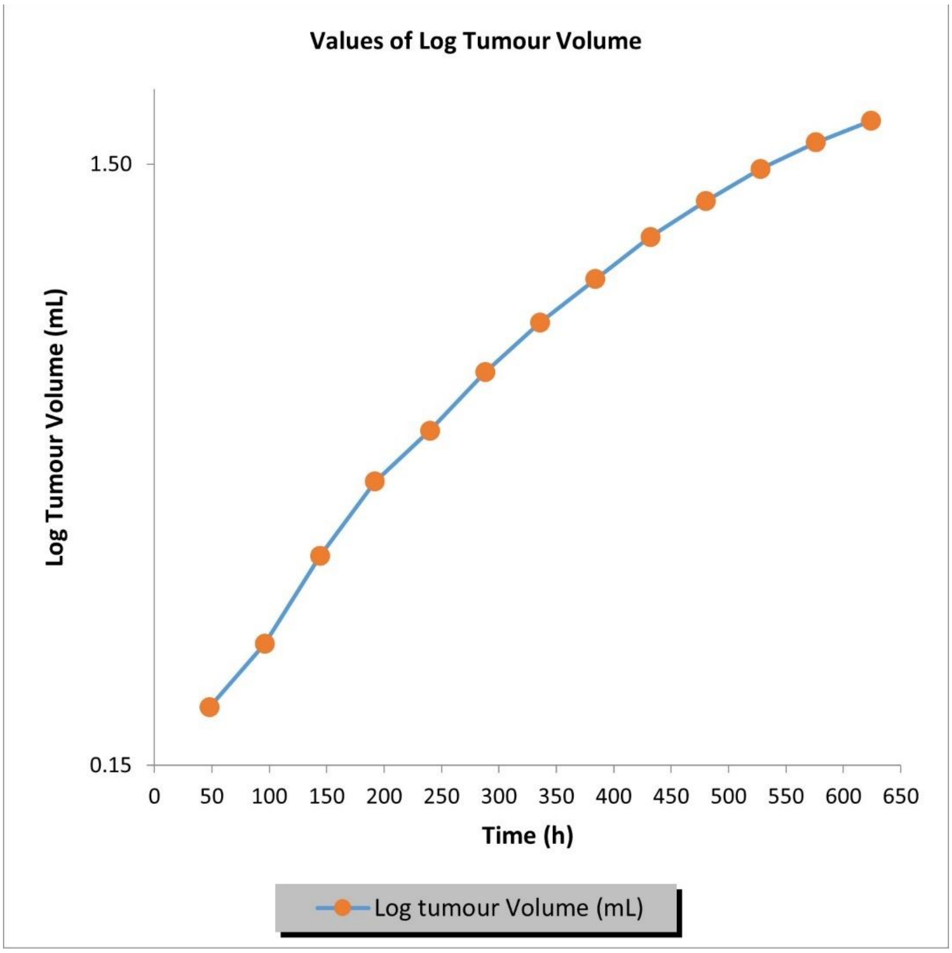

Figure S3. Logarithmic A375 tumour volume in time showing a clear trend of tumour growth saturation after vehicle administration. Observed data are means from $n = 11$.

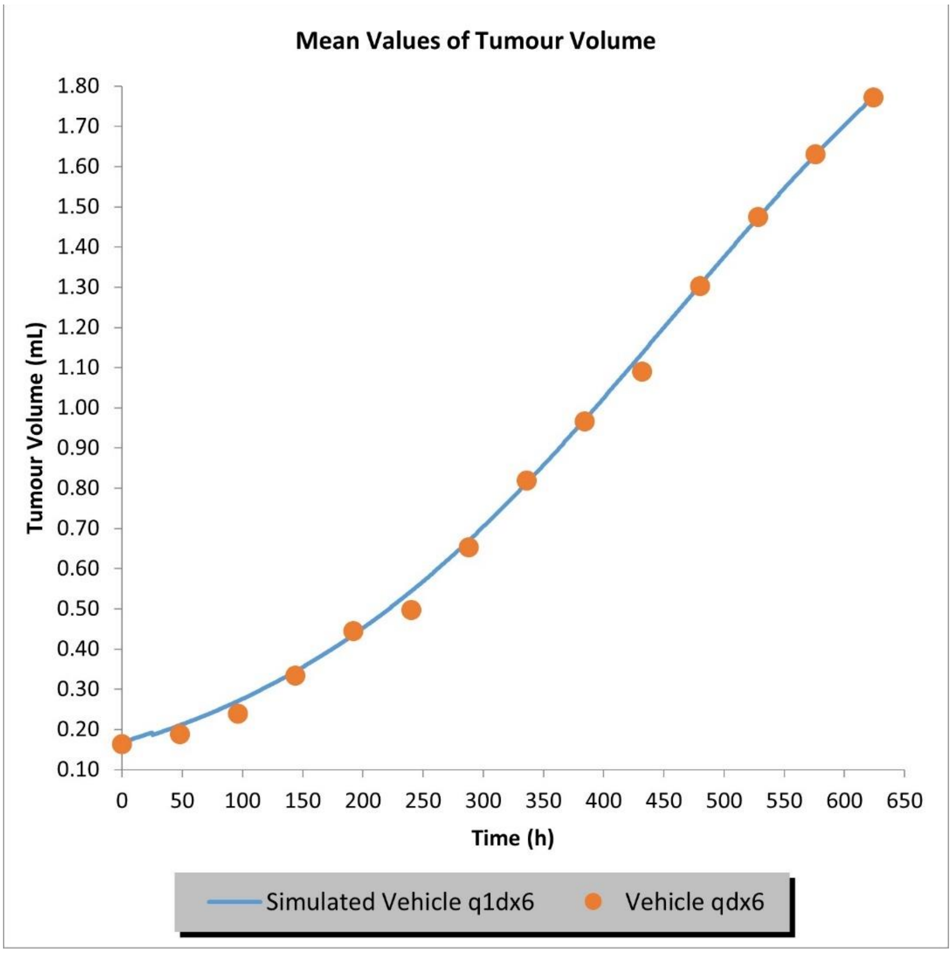

Figure S4. Logistic A375 tumour growth model, after vehicle administration. Observed data are means from n = 11.

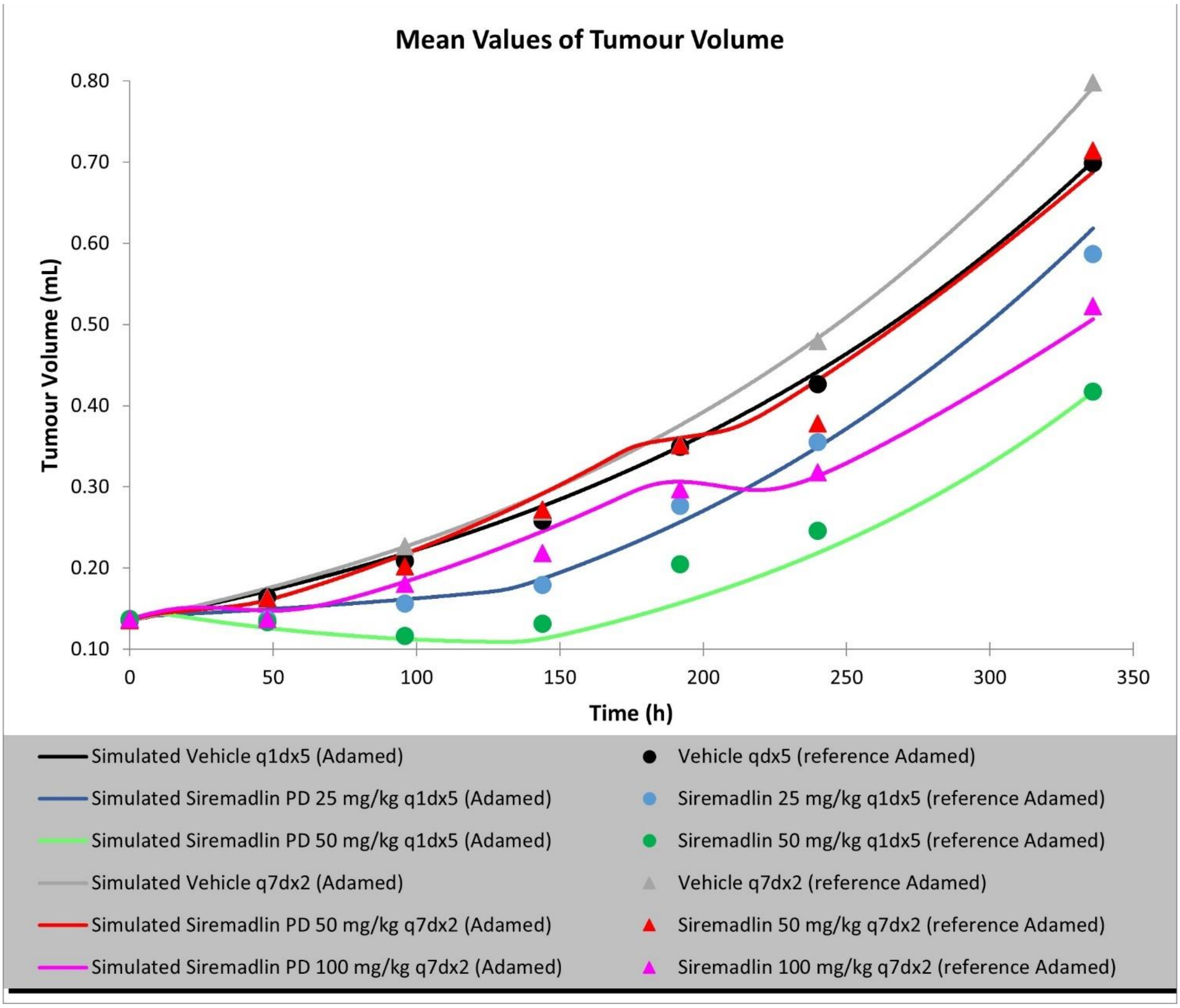

Figure S5. TGI models of siremadlin from Adamed Pharma verification dataset. Observed data are means from $n = 5$.

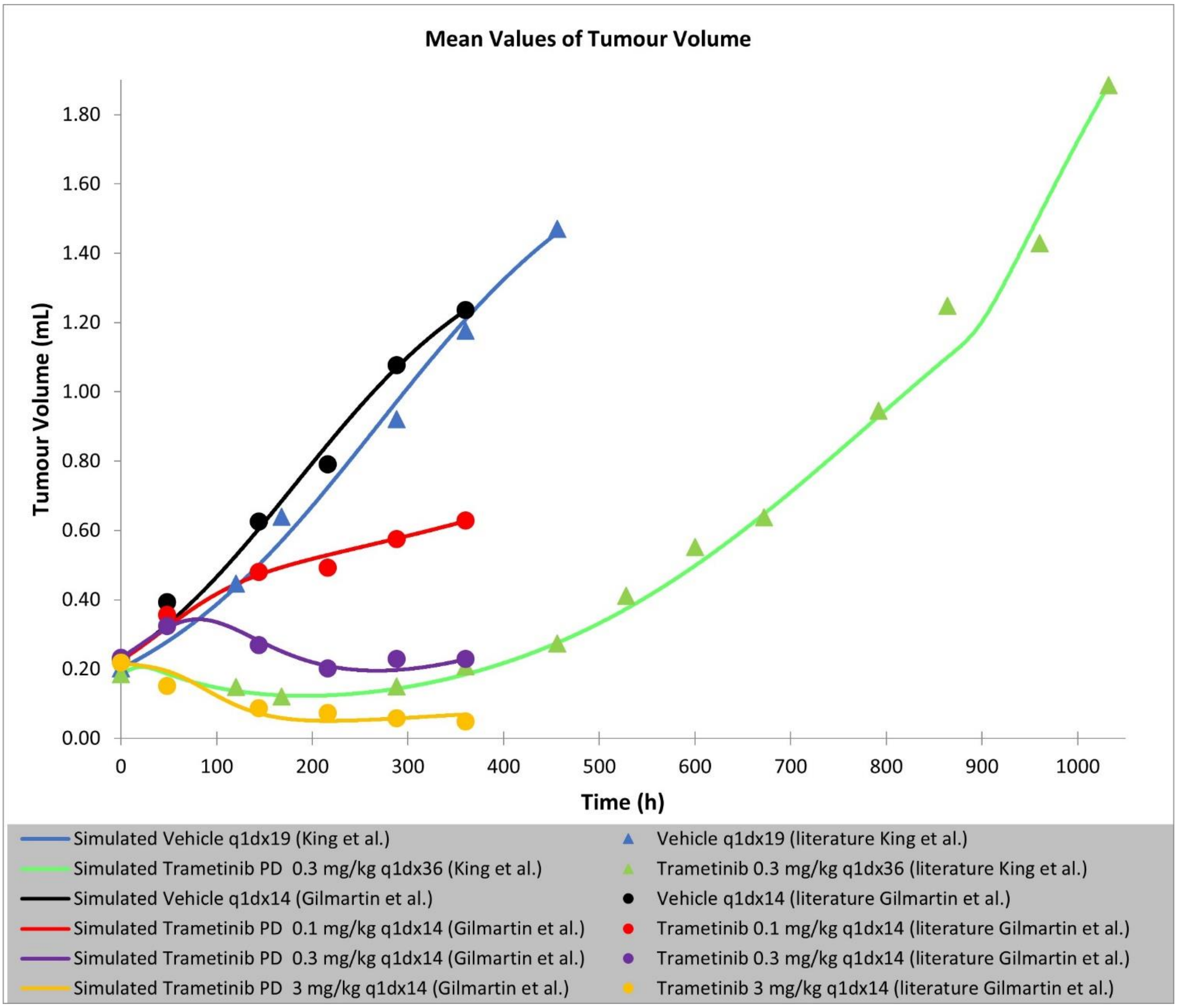

Figure S6. TGI models of trametinib from literature verification datasets: 0.3 mg/kg qdx36 (data digitized from King et al. [8]), 0.1/0.3/3 mg/kg qdx14 (data digitized from Gilmartin et al. [9]).

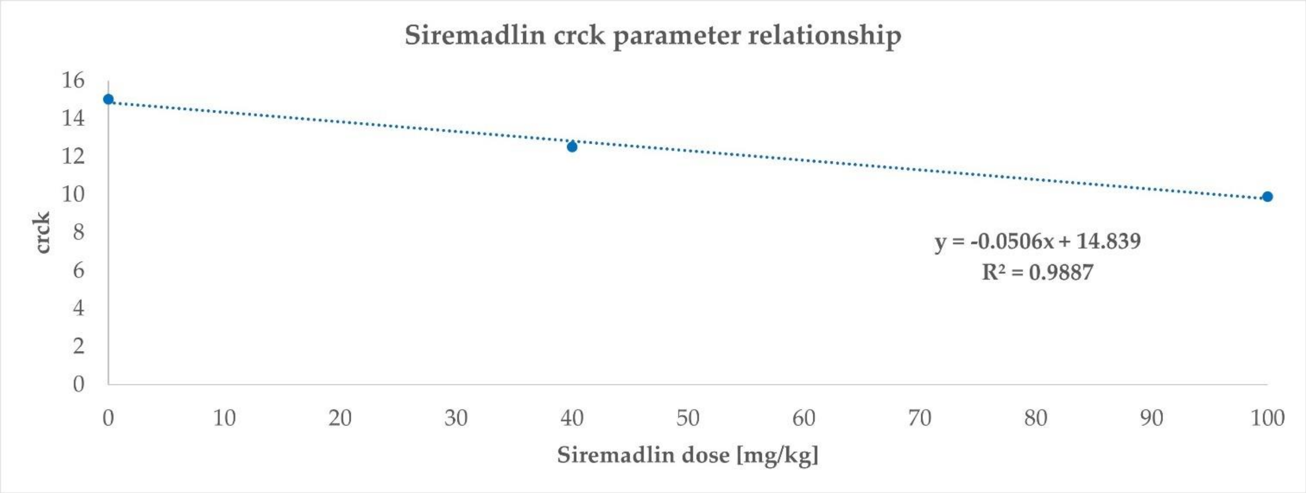

Figure S7. Siremadlin crck parameter relationship in universal TGI model.

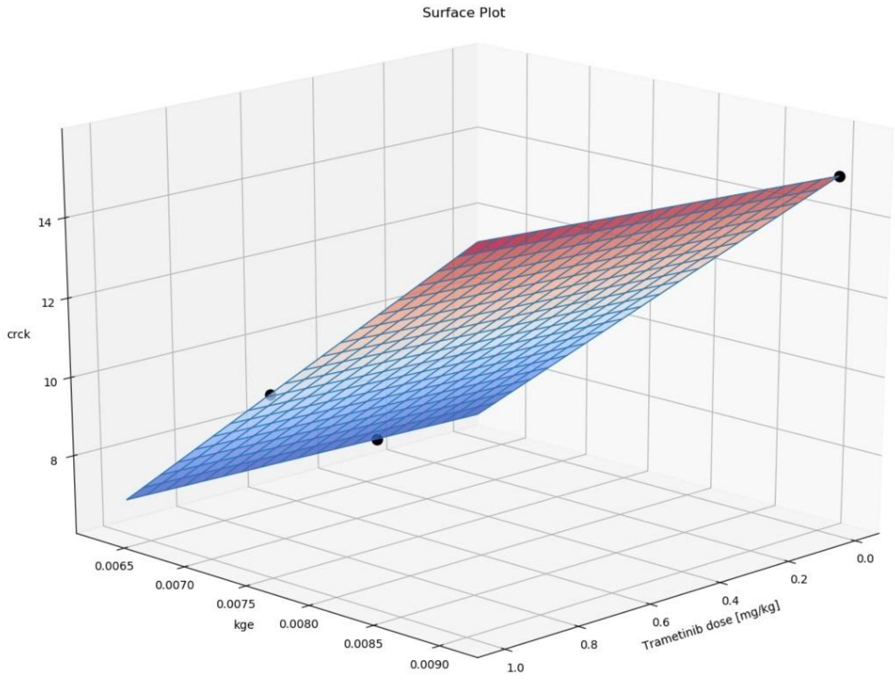

Figure S8. Trametinib crck parameter relationship in universal TGI model.

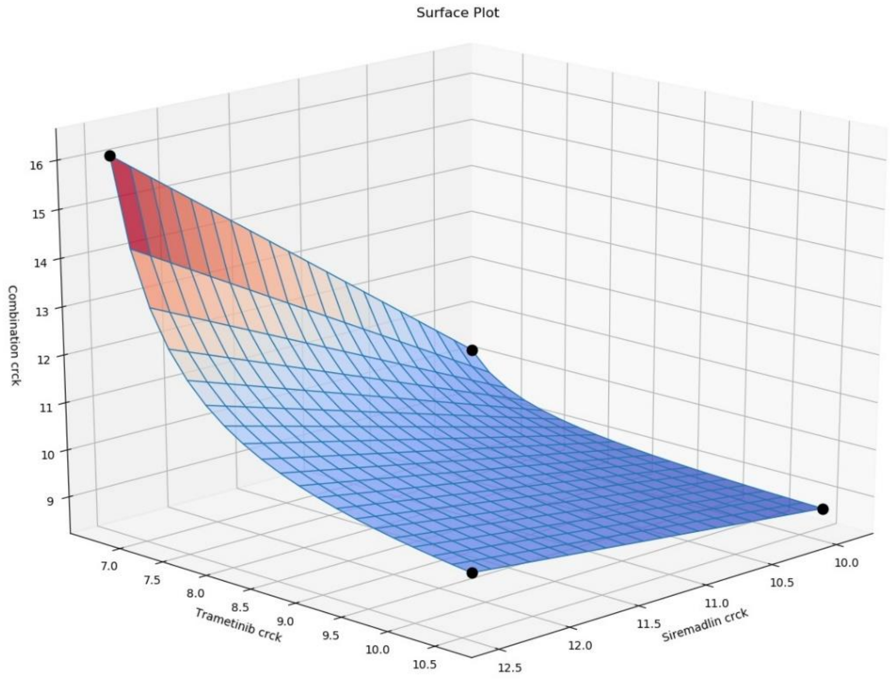

Figure S9. Siremadlin and trametinib combination crck parameter relationship in universal TGI model.

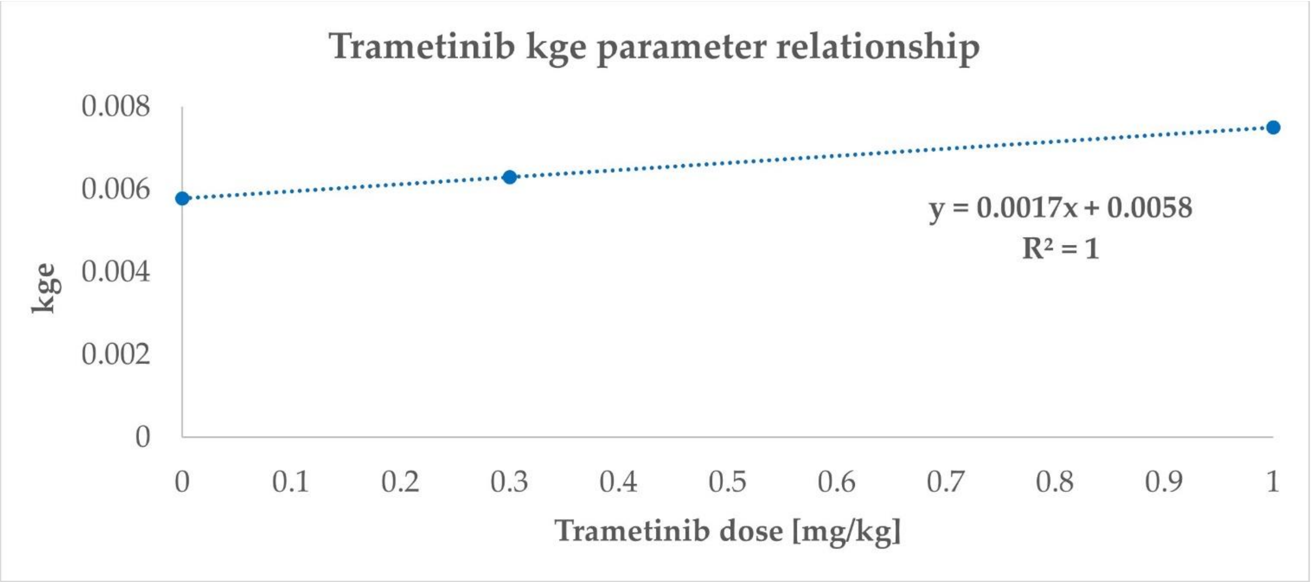

Figure S10. Trametinib kge parameter relationship in universal TGI model.

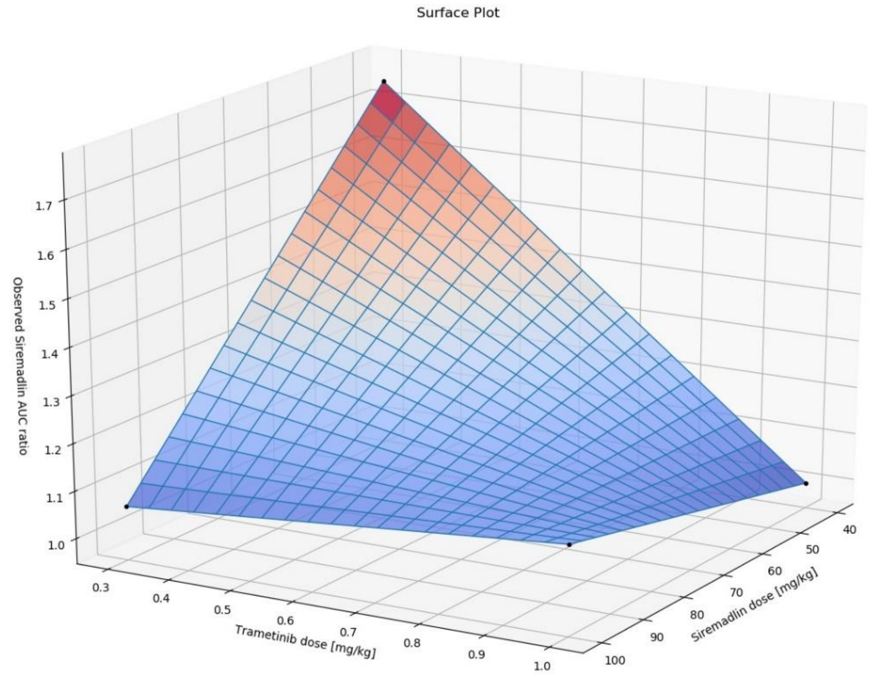

Figure S11. Siremadlin exposure ratio parameter relationship in universal TGI model.

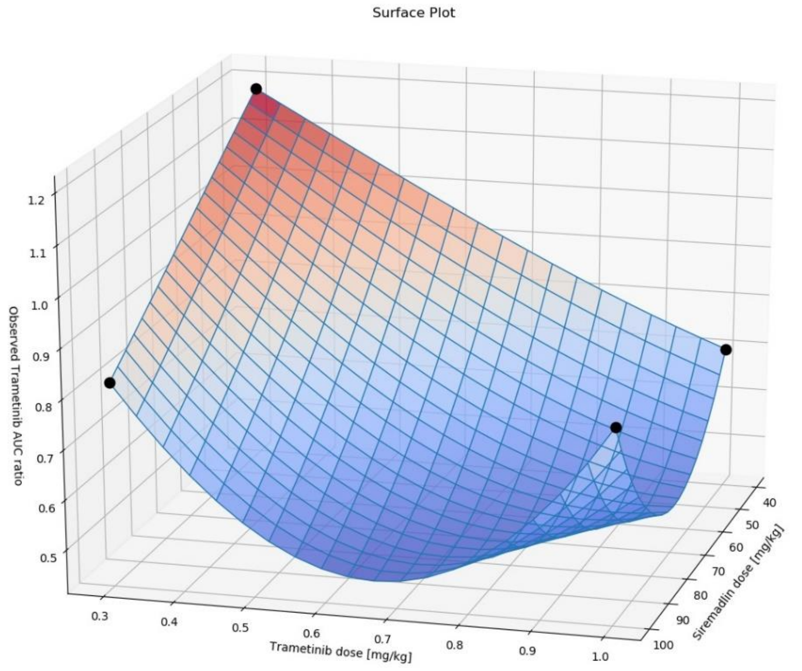

Figure S12. Trametinib exposure ratio parameter relationship in universal TGI model.

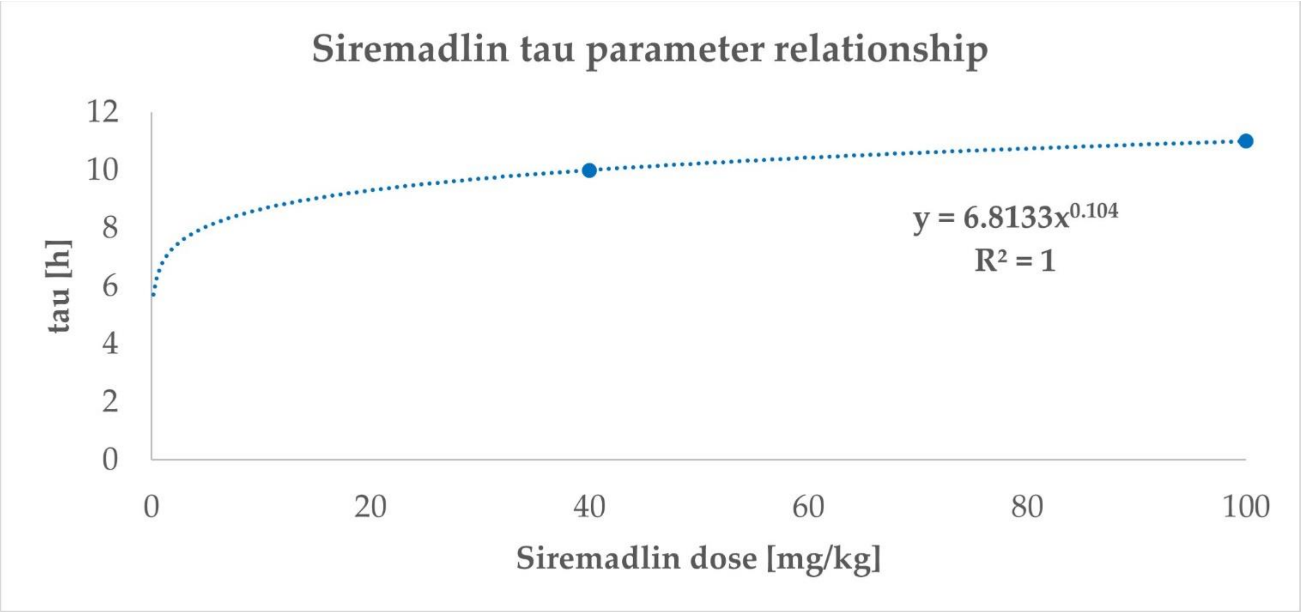

Figure S13. Siremadlin tau parameter relationship in universal TGI model.

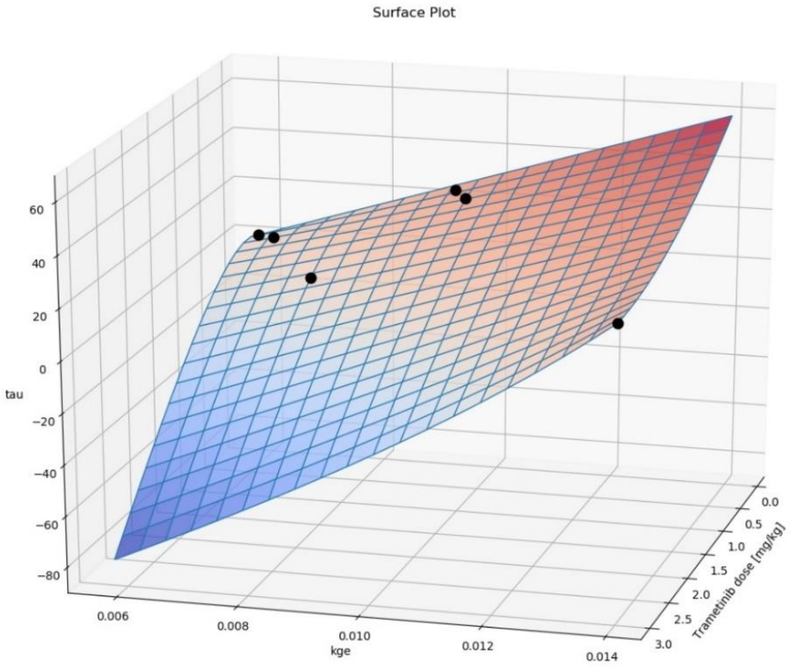

Figure S14. Trametinib tau parameter relationship in universal TGI model.

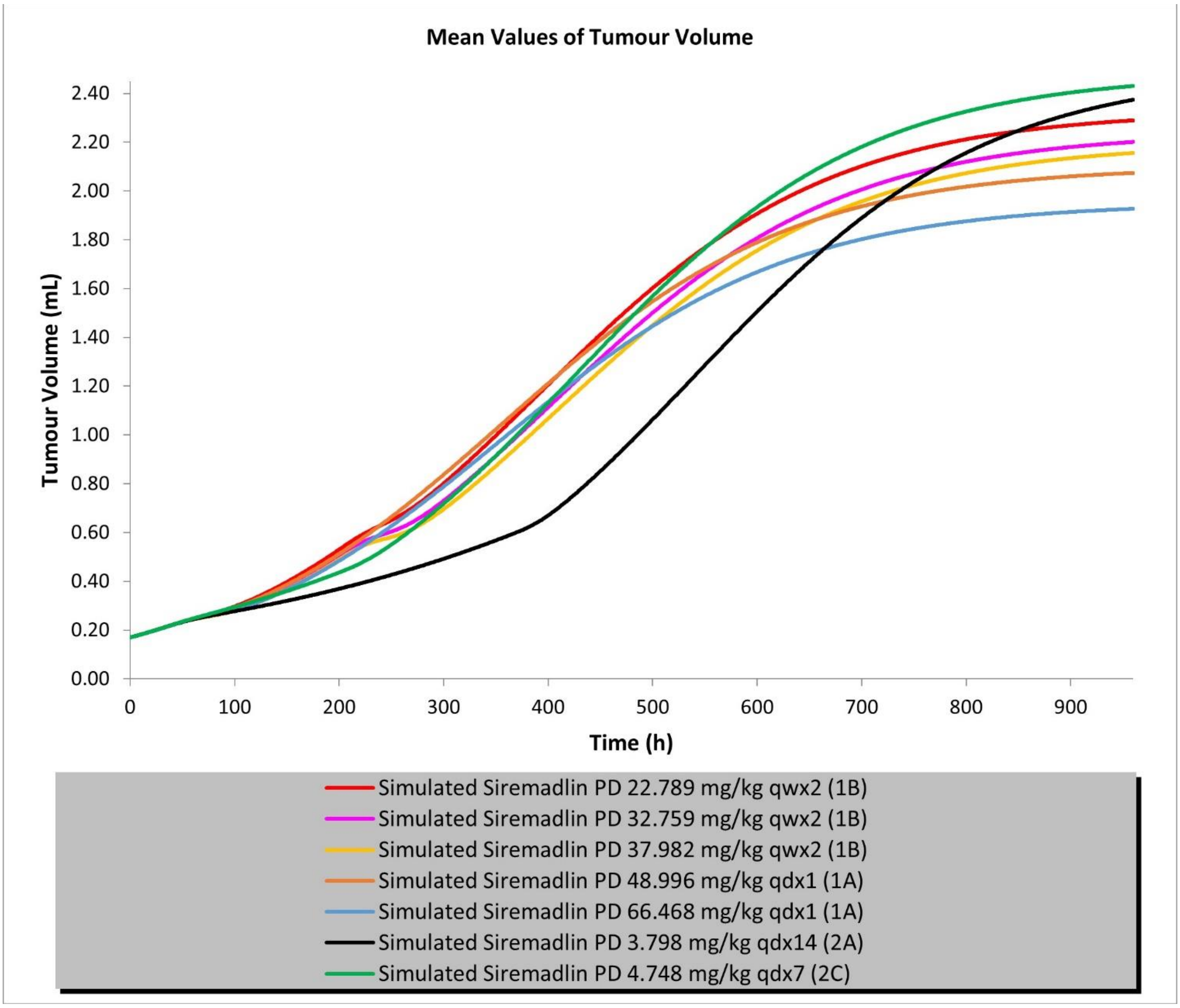

Figure S15. Simulation of siremadlin efficacy at HED doses (1 therapy cycle).

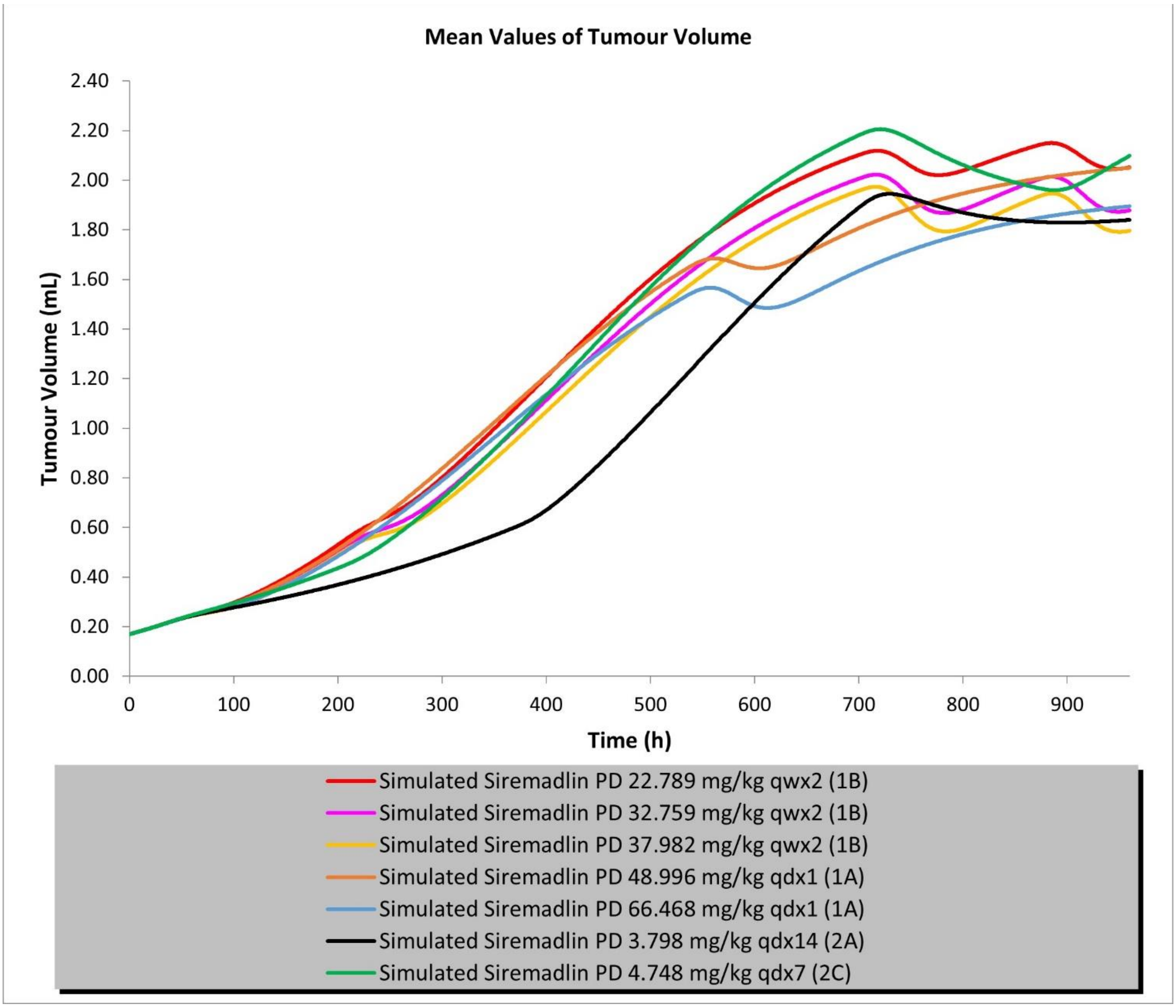

Figure S16. Simulation of siremadlin efficacy at HED doses (2 therapy cycles).

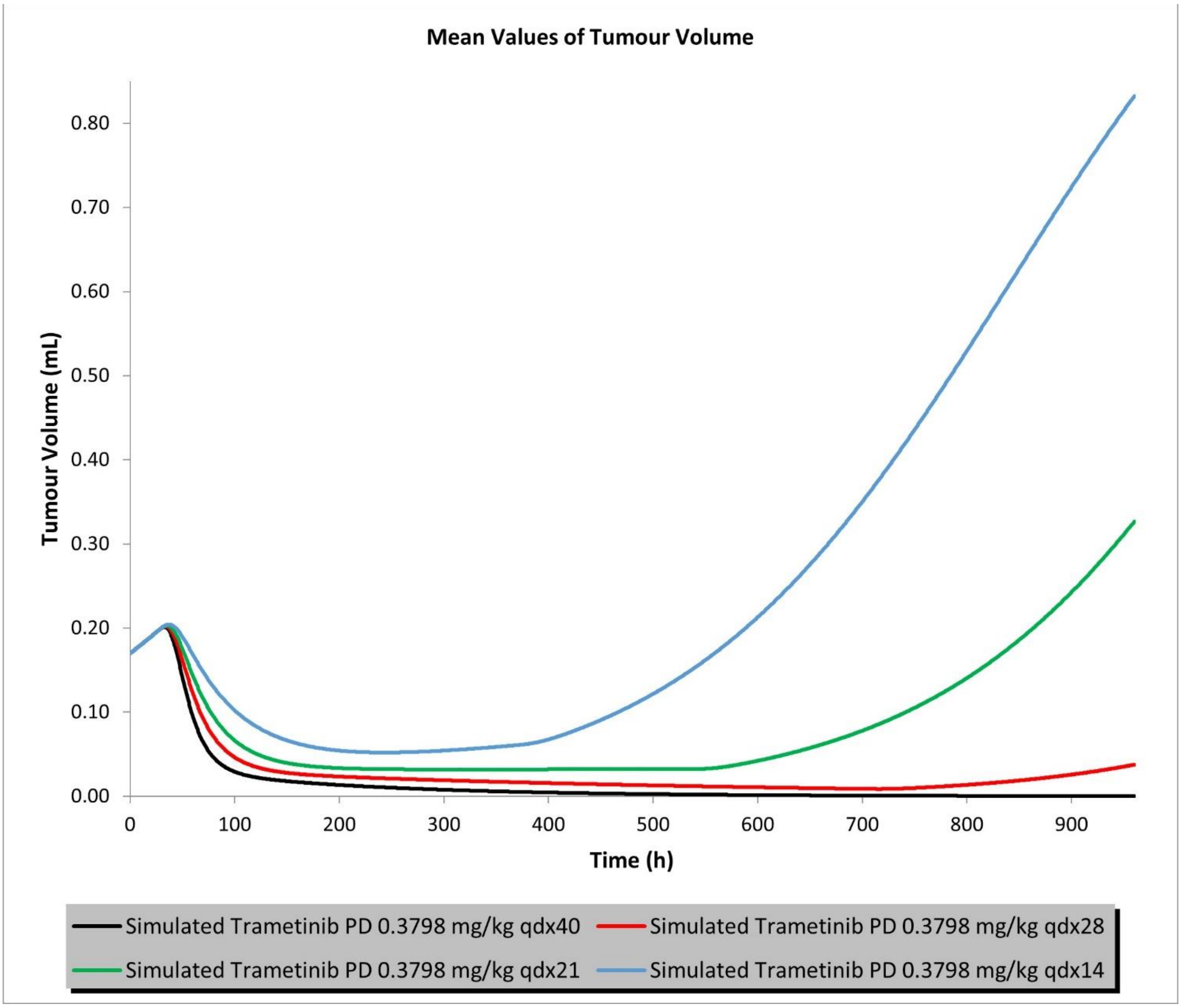

Figure S17. Simulation of trametinib efficacy at HED doses (1 therapy cycle).

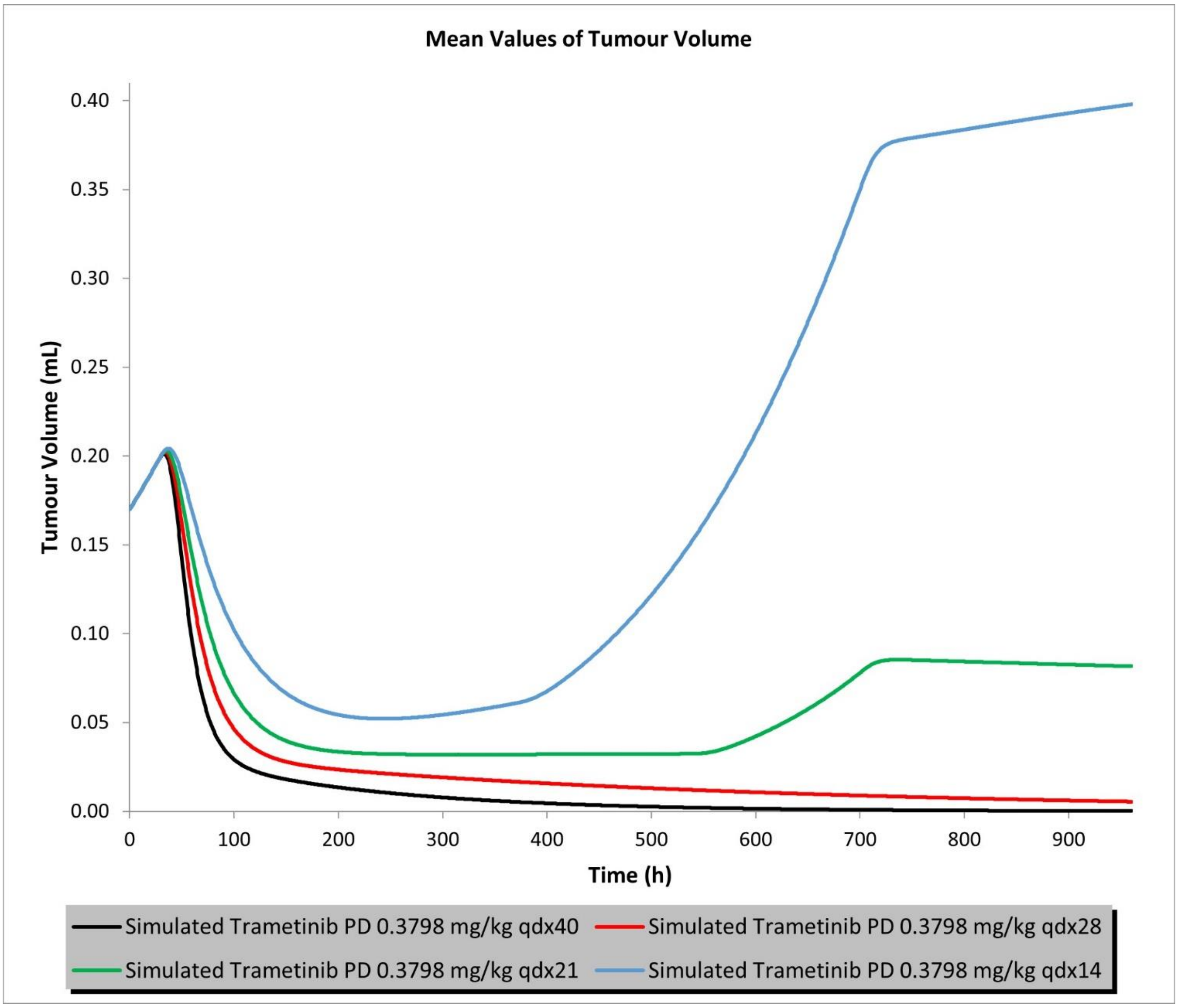

Figure S18. Simulation of trametinib efficacy at HED doses (2 therapy cycles).

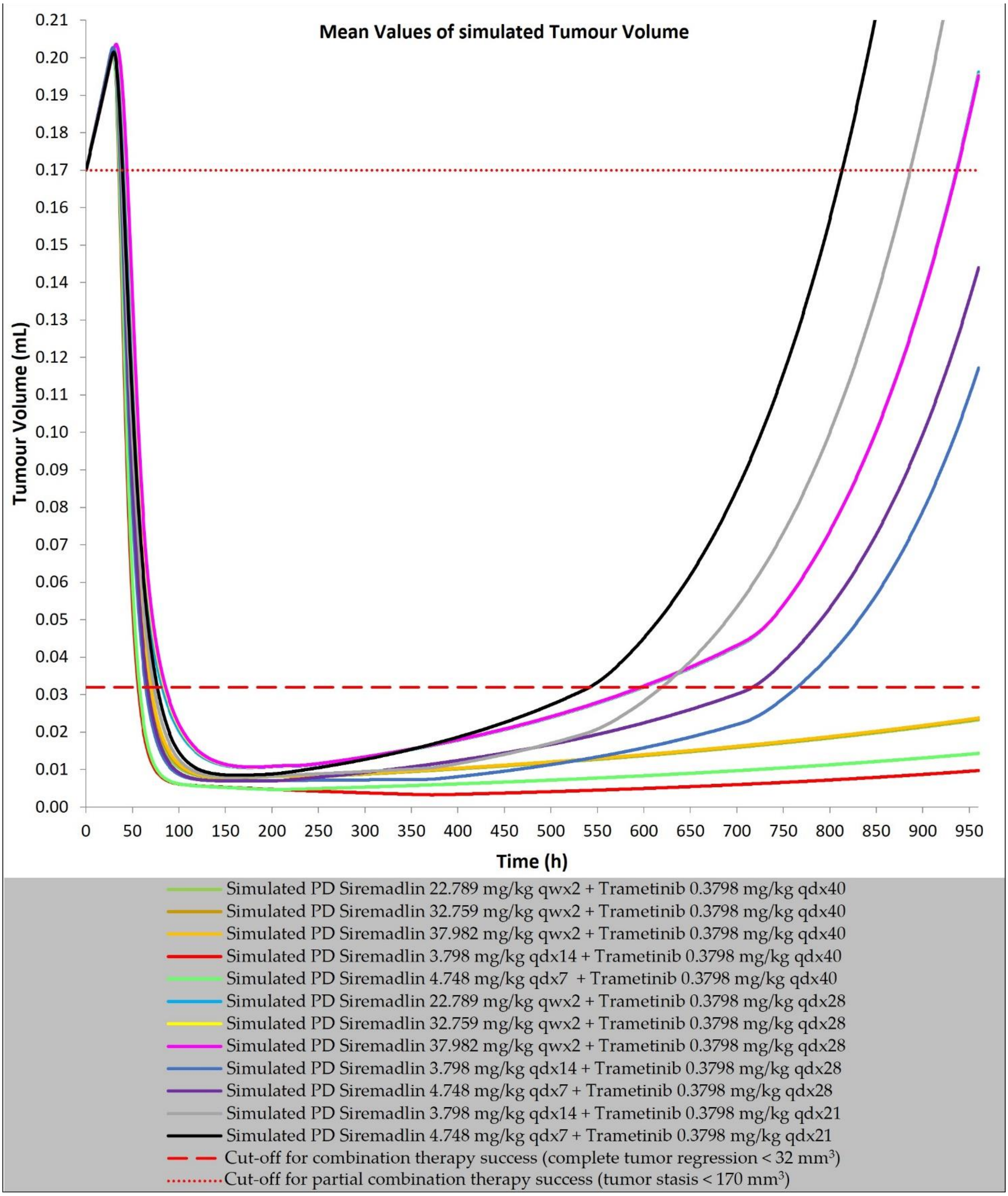

Figure S19. Simulation of siremadlin+trametinib combination efficacy at HED doses (1 therapy cycle).

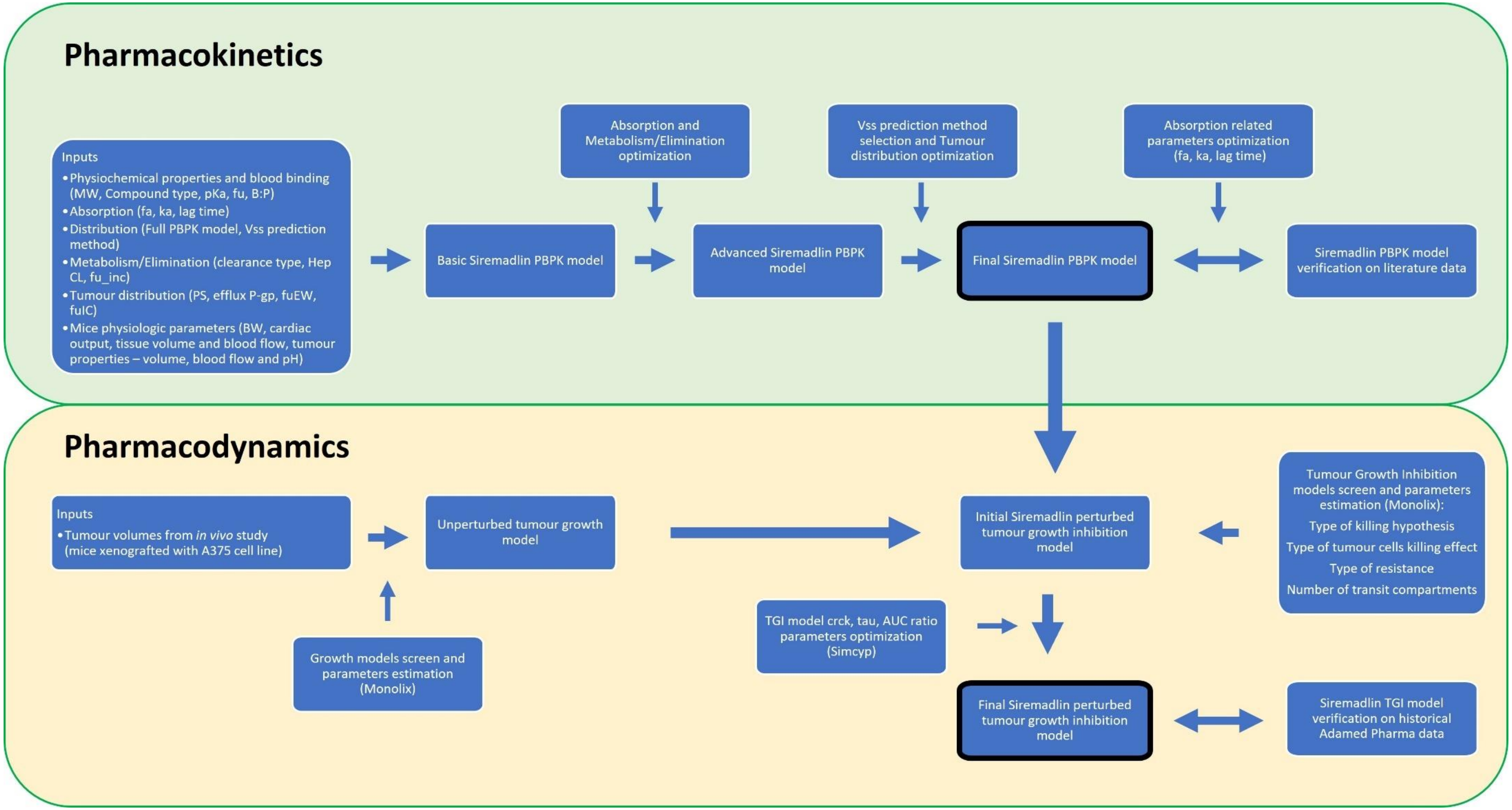

Figure S20. PBPK/PD model building workflow for siremadlin.

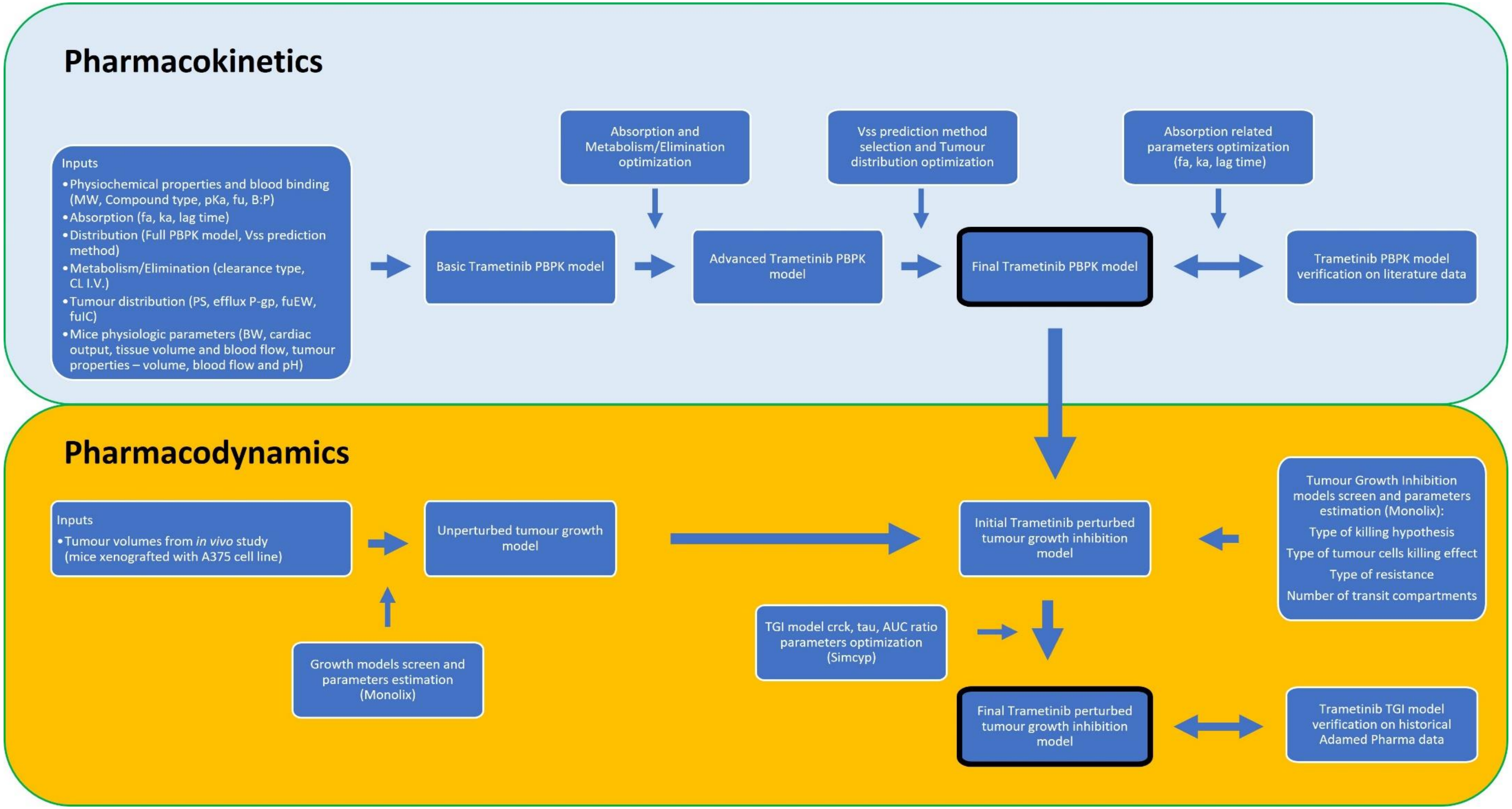

Figure S21. PBPK/PD model building workflow for trametinib.

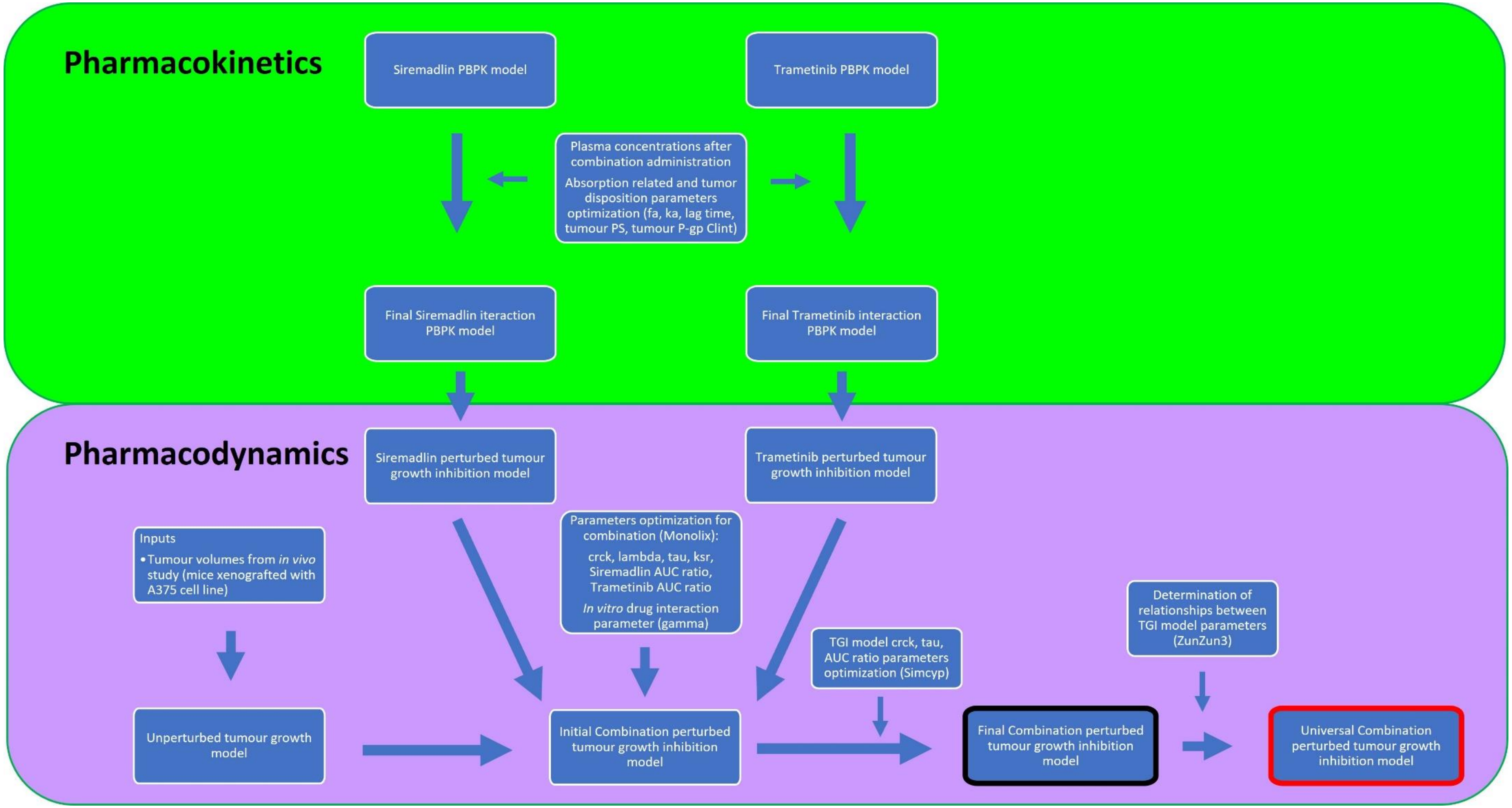

Figure S22. PBPK/PD model building workflow for siremadlin and trametinib drug combination.

Code S1. Mlxtran code for tumour growth inhibition model for drug combination (siremadlin + trametinib combination 100+1 mg/kg).

```
DESCRIPTION:Drug combination TGI model.
Model for TS (tumour size):
- Tumour growth follows a logistic model. This model assumes an exponential growth rate (kge) which decelerates linearly with respect to the tumour size. This results in sigmoidal dynamics - with an initial exponential growth phase followed by a growth-saturated phase as the tumour reaches its carrying capacity (TSmax). The initial tumour size is TS0.
Tumour growth inhibition model:
- Tumour growth inhibition model using a log-kill killing hypothesis where the treatment effect follows exponential kill kinetics (kkill*(1-exp(-s*EXPOSURE))).
- A delay in treatment effect has been added by the introduction of 4 signal transit compartments (K1, K2, K3, K4). The duration of this delay is determined by the parameter tau.
Treatment:
- The treatment effect is based on EXPOSURE, which is the pharmacokinetics of the treatment modelled using the pkmodel macro.

[LONGITUDINAL]
input = {TS0, TSr0, crck, kge, s, lambda, tau, ksr, gamma, AUC_ratio_Siremadlin, AUC_ratio_TRA}
TS0 = {use=regressor}

PK:
;Siremadlin (PK parameters assume PK interaction during the first 96h. PK estimates from 2 compartmental models including introduction of lag time, first order absorption process and linear clearance)

Tlag_Siremadlin = 0.000001
ka_Siremadlin = 0.88555
Cl_Siremadlin   = 0.0012175
V1_Siremadlin   = 0.002802
Q_Siremadlin    = 530.58255
V2_Siremadlin   = 0.003598
compartment(cmt = 1, volume=V1_Siremadlin, concentration=C_Siremadlin)
absorption(adm=1, cmt=1, ka=ka_Siremadlin, Tlag = Tlag_Siremadlin)
elimination(cmt=1, Cl=Cl_Siremadlin)
peripheral(k12=Q_Siremadlin/V1_Siremadlin, k21=Q_Siremadlin/V2_Siremadlin)

;TRAMETINIB (PK parameters assume PK interaction during the first 96h. PK estimates from 2 compartmental models including introduction of lag time, first order absorption process and linear clearance)

Tlag_TRA = 0.5122
ka_TRA = 0.6863
Cl_TRA    = 0.00023
```

```
V1_TRA    = 0.0009
Q_TRA     = 0.00002200
V2_TRA    = 0.00019
compartment(cmt = 3, volume=V1_TRA, concentration=C_TRA)
absorption(adm=2, cmt=3, ka=ka_TRA, Tlag = Tlag_TRA)
elimination(cmt=3, Cl=Cl_TRA)
peripheral(k34=Q_TRA/V1_TRA, k43=Q_TRA/V2_TRA)
;Exposure in drug combination
EXPOSURE = C_Siremadlin+C_TRA

EQUATION:
odeType=stiff
TSmax = crck*(TS0+TSr0)

TS_0 = TS0
TSr_0 = TSr0
K1_0 = 0
K2_0 = 0
K3_0 = 0
K4_0 = 0

kkill_Siremadlin = 0.0215
kkill_TRA = 0.0235
kkill_combined = (kkill_Siremadlin *AUC_ratio_Siremadlin + kkill_TRA *AUC_ratio_TRA)
if t>=96
    kkill = kkill_TRA
else
    kkill = kkill_combined * gamma
end

; Signal distribution
dK = (kkill*(1-exp(-s*EXPOSURE)))
ddt_K1 = (dK-K1)/tau
ddt_K2 = (K1-K2)/tau
ddt_K3 = (K2-K3)/tau
ddt_K4 = (K3-K4)/tau

TotalTS = TS+TSr

;Saturation for TS and TSr at 1e12 to avoid infinite values
if TS>1e12 | TSr>1e12
  TSDynamics = 0
```

```
  TSrDynamics = 0
else
  TSDynamics = (kge*TS*(1-(TotalTS/TSmax))) - (K4+ksr*K4)*TS
  TSrDynamics = (kge*TSr*(1-(TotalTS/TSmax))) + (ksr*K4*TS) - (K4/lambda*TSr)
end

ddt_TS = TSDynamics ; Treatment-sensitive cell population
ddt_TSr = TSrDynamics ; Treatment-resistant cell population

OUTPUT:
output = {TotalTS}
```

Code S2. Lua code for the drug interaction model (siremadlin + trametinib combination 100+1 mg/kg).

```
function popSimSetup(...)
      return 0
end

function odeInitStep(xin, su, P, ...)
      return 0
end

function odeRateStep(t, xin, su, gu, P, ...)
--Substrate (Siremadlin) properties with no PK interaction
local faSub = 0.60231                               -- substrate fraction absorbed (%)
local kaSub =  0.31                                 -- substrate absorption rate constant (1/h)
--Metabolite (Trametinib) properties with no PK interaction
local faMet = 0.7247                                -- metabolite fraction absorbed (%)
local kaMet = 1.25                                  -- metabolite absorption rate constant (1/h)
local tlagMet = 0.85                                -- metabolite lag time (h)
--Dosing schedules
local DosingTimesSubstrate = {24, 48, 72}           -- substrate dosing qdx3 (h)
local DosingTimesMetabolite = {24, 48, 72, 96, 120, 144}   -- metabolite dosing qdx6 (h)
local CoadministrationTimes = {24, 48, 72}          -- co-administration times (h)

for i=1,3
do
if (t>=CoadministrationTimes[i] and t<=96)
then
   --Substrate (Siremadlin) properties with PK interaction
   kaSub = 0.65
   --Metabolite (Trametinib) properties with PK interaction
   faMet = 0.6407
   kaMet = 0.357
   tlagMet = 0
end

local doseSub = 4.96029960              --Siremadlin 100 mg/kg dose in uM adjusted to mice mass 27.55g
local doseMet = 0.04476836              --Trametinib 1 mg/kg dose in uM adjusted to mice mass 27.55g
local vLiv = 0.00119                    --mouse liver volume in (L)
local Qpv = 0.16578072                  --portal vein blood flow l/h
```

```
local BPsub =  0.76                        --Siremadlin blood to plasma ratio
local BPmet = 0.7                          --Trametinib blood to plasma ratio
local RateInSub =  0                       --Substrate dosing rate (uM/h)
local RateInMet = 0                        --Metabolite dosing rate (uM/h)

for i=1,3 --substrate dosing according to the schedule
do
if (t>DosingTimesSubstrate[i])
then
RateInSub = RateInSub + doseSub*kaSub*faSub*math.exp(- kaSub * (t-DosingTimesSubstrate[i]))
end
end

for j=1,6 --metabolite dosing according to the schedule
do
if (t>(DosingTimesMetabolite[j]+tlagMet))
then
RateInMet = RateInMet + doseMet*kaMet*faMet*math.exp(- kaMet * (t-tlagMet-DosingTimesMetabolite[j]))
end
end

local C_Pv = RateInSub/(Qpv*BPsub)                               --substrate input into the portal vein
local C_Pv_met = RateInMet/(Qpv*BPmet)                           --metabolite input into the portal vein

local SubsysGradient = sc:getGradient(0)                         --substrate liver state variable index
local newSubsysGradient = SubsysGradient + (Qpv*C_Pv*BPsub/vLiv) --substrate input into liver

local MetsysGradient = sc:getGradient(21)                        --metabolite liver state variable index
local newMetsysGradient = MetsysGradient + (Qpv*C_Pv_met*BPmet/vLiv)--metabolite input into liver

sc:setGradient(0,newSubsysGradient)
sc:setGradient(21,newMetsysGradient)

   return C_Pv
end
end
```

Code S3. Lua code for tumour growth inhibition model for siremadlin + trametinib combination 100+1 mg/kg).

```
--Drug Combination Custom PD TGI model features:
--Tumour growth follows a logistic model
--Killing hypothesis: Log-kill killing hypothesis with exponential kill kinetics
--Delay in treatment effect (Signal distribution model of delay - Lobo & Balthasar 2002) using 4 transit compart-
ments
--Modeling of acquired resistance (2 subpopulations: sensitive and resistant)
--initial parameters estimates taken from models previously built in Monolix

function popSimSetup(...)
        sc:setParameterName(1, "Ts0")          --tumour size of cancer sensitive cells population (mL)
        sc:setParameterName(2, "Tsr0")         --tumour size of cancer resistant cells population (mL)
        sc:setParameterName(3, "crck")         --tumour size and Tsmax correlation constant
        sc:setParameterName(4, "kge")          --tumour growth (1/day)
        sc:setParameterName(5, "s")            --cancer sensitive cells population killing constants co-
efficient
        sc:setParameterName(6, "lambda")       --resistance factor (kkillr/kkill)
```

```
        sc:setParameterName(7, "tau")                   --killing effect delay (h)
        sc:setParameterName(8, "ksr")                   --sensitive to resistant cells conversion rate (%)
        sc:setParameterName(9, "gamma")                 --PD interaction parameter taken from in vitro (%)
        sc:setParameterName(10, "TSmax")                --maximal tumour size (mL)
        sc:setParameterName(11, "kkillTrametinib")      --killing effect constant for Trametinib
        sc:setParameterName(12, "kkillSiremadlin")          --killing effect constant for Siremadlin
        sc:setParameterName(13, "AUCratioSiremadlin")       --exposure ratio constant for Siremadlin in com-
bination (PK DDI with Trametinib)
        sc:setParameterName(14, "AUCratioTrametinib")--exposure ratio constant for Trametinib in combina-
tion (PK DDI with Siremadlin)

end


function individualSetup(...)
    --parameters initial estimates from Monolix
  local Ts0  = 0.1681                     --tumour size of cancer sensitive cells population (mL)
  local Tsr0  = 0.000001                  --tumour size of cancer resistant cells population (mL)
  local crck  = 8.75                      --tumour size and TSmax correlation constant
  local kge  = 0.0075                     --tumour growth (1/day)
  local s  = 0.3                          --cancer sensitive cells population killing constants coefficient
  local lambda  = 12                      --resistance factor (kkillr/kkill)
  local tau  = 10.5                       --killing effect delay (h)
  local ksr  = 0.0302445                  --sensitive to resistant cells conversion rate (%)
  local gamma  = 1.2313                   --PD interaction parameter taken from in vitro (%)
  local kkillSiremadlin = 0.0215          --killing effect constant for Siremadlin
  local kkillTrametinib = 0.0235          --killing effect constant for Trametinib
  local AUCratioSiremadlin = 1.1357           --AUC ratio for Siremadlin PK DDI
  local AUCratioTrametinib = 0.80365      --AUC ratio for Trametinib PK DDI
  local TumourPSSubNI = 0.084656          --Substrate (Siremadlin) passive permeability clearance between
intra- and extracellular water of tumour (No Interaction) – mL/min/mL
  local TumourCLSubNI = 0.09554           --Substrate (Siremadlin) in vitro transporter mediated intrinsic
clearance in tumour (No Interaction) – mL/min/mL
  local TumourPSMetNI = 0.02482           --Metabolite (Trametinib) passive permeability clearance between
intra- and extracellular water of tumour (No Interaction) – mL/min/mL
  local  TumourCLMetNI  =  0.06027        --Metabolite (Trametinib) in vitro transporter mediated intrinsic
clearance in tumour (No Interaction) – mL/min/mL
  local TumourPSSubI = 0.1407             --Substrate (Siremadlin) passive permeability clearance between
intra- and extracellular water of tumour (Interaction) – mL/min/mL
  local TumourCLSubI = 0.04712            --Substrate (Siremadlin) in vitro transporter mediated intrinsic
clearance in tumour (Interaction) – mL/min/mL
  local TumourPSMetI = 0.11092            --Metabolite (Trametinib) passive permeability clearance between
intra- and extracellular water of tumour (Interaction) – mL/min/mL
  local TumourCLMetI = 0.21443            --Metabolite (Trametinib) in vitro transporter mediated intrinsic
clearance in tumour (Interaction) – mL/min/mL
  local PSScalarSub = 1.662               --TumourPSSubI/TumourPSSubNI
  local TumourCLScalarSub = 0.493         --TumourCLSubI/TumourCLSubNI
  local PSScalarMet = 4.469               --TumourPSMetI/TumourPSMetNI
  local TumourCLScalarMet = 3.558         --TumourCLMetI/TumourCLMetNI

     sc:setParameter(1,  Ts0)
    sc:setParameter(2,  Tsr0)
    sc:setParameter(3,  crck)
    sc:setParameter(4,  kge)
```

```
        sc:setParameter(5,  s)
        sc:setParameter(6,  lambda)
        sc:setParameter(7,  tau)
        sc:setParameter(8,  ksr)
        sc:setParameter(9,  gamma)
        sc:setParameter(10, TSmax)
        sc:setParameter(11, kkillTrametinib)
        sc:setParameter(12, kkillSiremadlin)
        sc:setParameter(13, AUCratioSiremadlin)
        sc:setParameter(14, AUCratioTrametinib)
end


function odeInitStep(su, P, ...)
      -- delay transit compartments
   su[1]  = 0               --  K1 first transit compartment
   su[2]  = 0               --  K2 second transit compartment
   su[3]  = 0               --  K3 third transit compartment
   su[4]  = 0               --  K4 fourth transit compartment
   su[5]  = 0.1681          -- tumour size of cancer sensitive cells population estimate (mL)
   su[6] = 0.000001         -- tumour size of cancer resistant cells population estimate (mL)
   return 0
end

function odeRateStep(t,su,gu,P,...)

   C_Siremadlin = 1000 *     sc:getIndivPlasmaConc(sc.SUBSTRATE)    -- get substrate (Siremadlin) systemic concentrations (units conversion uM --> nM)
   C_Trametinib = 1000 * sc:getIndivPlasmaConc(sc.PRIMET1SUB)    -- get metabolite (Trametinib) systemic concentrations (units conversion uM --> nM)

   --assign particular parameters to the array
   local Ts0 =                          P[1]
    local Tsr0 =                        P[2]
    local crck =                        P[3]
    local kge =                         P[4]
    local s =                           P[5]
    local lambda =                      P[6]
    local tau =                         P[7]
    local ksr =                         P[8]
    local gamma =                       P[9]
    local TSmax =                       P[10]
    local kkillTrametinib =             P[11]
    local kkillSiremadlin =             P[12]
    local AUCratioSiremadlin =              P[13]
    local AUCratioTrametinib =          P[14]

      local TSmax = crck*(Ts0+Tsr0) -- maximal tumour volume based on initial tumour size and maximal tumour volume correlation constant

   --Drug interaction at the tumour distribution level, substrate and metabolite tumour PS and CL scaled. Disabled to speed up calculation time.
   if t>=24 and t<=96
   then
```

```
--sc:feedbackIndivTumourVasPSScalar(sc.SUBSTRATE, 1.662)
--sc:feedbackIndivTumourCLScalar(sc.SUBSTRATE, 0.493)
--tumour interaction for metabolite available if drug distribution is set as minimal PBPK model instead full PBPK model
--sc:feedbackIndivTumourVasPSScalar(sc.PRIMET1SUB, 4.469)
--sc:feedbackIndivTumourCLScalar(sc.PRIMET1SUB, 3.558)
end

local kkill = kkillTrametinib --Basic killing effect is assigned to Trametinib since it is longer administered

--Pharmacodynamic interaction at the tumour killing effect for drug combination assuming PD interaction parameter taken from in vitro studies and differences in exposure for both drugs
if t>=24 and t<=96
then
kkill = (kkillSiremadlin*AUCratioSiremadlin + kkillTrametinib*AUCratioTrametinib)*gamma
  end

--killing effect is assigned to the concentrations of both drugs in plasma
local TK = (kkill*(1-math.exp(-s*(C_Siremadlin+C_Trametinib))))
  --Delay of effect for sensitive cancer population by transit compartments
  gu[1] = (TK -su[1])      / tau
  gu[2] = (su[1] - su[2]) / tau
  gu[3] = (su[2] - su[3]) / tau
  gu[4] = (su[3] - su[4]) / tau

  TTS = su[5] + su[6] --initial total tumour size
-- To avoid infinite values of tumour volumes saturation of tumour growth for TS and TSr at 1e12 volume was introduced
if (su[5] > 1E12) or (su[6] > 1E12) then
   gu[5]  =  0
   gu[6] = 0
else
   gu[5] = (kge*su[5]*(1-(TTS/TSmax))) - (su[4]+ksr*su[4])*su[5]                    --change tumour volume of treatment sensitive cancer cells population
   gu[6] = (kge*su[6]*(1-(TTS/TSmax))) + (ksr*su[4]*su[5]) - ((su[4]/lambda)*su[6])     --change tumour volume of treatment resistant cancer cells population
end

  TS =  su[5]
  TSr = su[6]

  TotalTumourSize = TS + TSr --total tumour size

  sc:feedbackTumourVol(TotalTumourSize)
  return TotalTumourSize --tumour size output
end
```

References

1. Witkowski, J.; Polak, S.; Rogulski, Z.; Pawelec, D. In Vitro/In Vivo Translation of Synergistic Combination of MDM2 and MEK Inhibitors in Melanoma Using PBPK/PD Modelling: Part I. *International Journal of Molecular Sciences* **2022**, *Submitted*.

2. Jeay, S.; Ferretti, S.; Holzer, P.; Fuchs, J.; Chapeau, E.A.; Wartmann, M.; Sterker, D.; Romanet, V.; Murakami, M.; Kerr, G.; et al. Dose and Schedule Determine Distinct Molecular Mechanisms Underlying the Efficacy of the P53–MDM2 Inhibitor HDM201. *Cancer Research* **2018**, *78*, 6257–6267, doi:10.1158/0008-5472.CAN-18-0338.
3. Lei, Y.; Zhenglin, Y.; Heng, L. MDM2 Inhibitors. U.S. Patent 11,339,171, 24 May 2022.
4. Yoo, J.Y.; Swanner, J.; Otani, Y.; Nair, M.; Park, F.; Banasavadi-Siddegowda, Y.; Liu, J.; Jaime-Ramirez, A.C.; Hong, B.; Geng, F.; et al. Oncolytic HSV Therapy Increases Trametinib Access to Brain Tumors and Sensitizes Them in Vivo. *Neuro Oncol* **2019**, *21*, 1131–1140, doi:10.1093/neuonc/noz079.
5. Pharmaceuticals and Medical Devices Agency (PMDA), Mekinist Initial Approval. Available online: https://www.pmda.go.jp/files/000233741.pdf (accessed on 19 August 2022).
6. Hofmann, F. Small Molecule HDM201 Inhibitor HDM201. In Proceedings of the AACR Annual Meeting 2016, New Orleans, LA, USA, 16–20 April 2016.
7. Food and Drug Administration (FDA), Trametinib Pharmacology Review. Available online: https://www.accessdata.fda.gov/drugsatfda_docs/nda/2013/204114Orig1s000PharmR.pdf (accessed on 19 August 2022).
8. King, A.J.; Arnone, M.R.; Bleam, M.R.; Moss, K.G.; Yang, J.; Fedorowicz, K.E.; Smitheman, K.N.; Erhardt, J.A.; Hughes-Earle, A.; Kane-Carson, L.S.; et al. Dabrafenib; Preclinical Characterization, Increased Efficacy When Combined with Trametinib, While BRAF/MEK Tool Combination Reduced Skin Lesions. *PLoS One* **2013**, *8*, e67583, doi:10.1371/journal.pone.0067583.
9. Gilmartin, A.G.; Bleam, M.R.; Groy, A.; Moss, K.G.; Minthorn, E.A.; Kulkarni, S.G.; Rominger, C.M.; Erskine, S.; Fisher, K.E.; Yang, J.; et al. GSK1120212 (JTP-74057) Is an Inhibitor of MEK Activity and Activation with Favorable Pharmacokinetic Properties for Sustained in Vivo Pathway Inhibition. *Clin Cancer Res* **2011**, *17*, 989–1000, doi:10.1158/1078-0432.CCR-10-2200.
10. Stein, E.M.; DeAngelo, D.J.; Chromik, J.; Chatterjee, M.; Bauer, S.; Lin, C.-C.; Suarez, C.; de Vos, F.; Steeghs, N.; Cassier, P.A.; et al. Results from a First-in-Human Phase I Study of Siremadlin (HDM201) in Patients with Advanced Wild-Type TP53 Solid Tumors and Acute Leukemia. *Clin Cancer Res* **2022**, *28*, 870–881, doi:10.1158/1078-0432.CCR-21-1295.
11. Flaherty, K.T.; Robert, C.; Hersey, P.; Nathan, P.; Garbe, C.; Milhem, M.; Demidov, L.V.; Hassel, J.C.; Rutkowski, P.; Mohr, P.; et al. Improved Survival with MEK Inhibition in BRAF-Mutated Melanoma. *New England Journal of Medicine* **2012**, *367*, 107–114, doi:10.1056/NEJMoa1203421.
12. Guerreiro, N.; Jullion, A.; Ferretti, S.; Fabre, C.; Meille, C. Translational Modeling of Anticancer Efficacy to Predict Clinical Outcomes in a First-in-Human Phase 1 Study of MDM2 Inhibitor HDM201. *AAPS J* **2021**, *23*, 28, doi:10.1208/s12248-020-00551-z.
